# Tnpo3 controls splicing of the pre-mRNA encoding the canonical TCR α chain of iNKT cells

**DOI:** 10.1038/s41467-023-39422-4

**Published:** 2023-06-20

**Authors:** Norimasa Iwanami, Andreas S. Richter, Katarzyna Sikora, Thomas Boehm

**Affiliations:** 1 Department of Developmental Immunology, Max Planck Institute of Immunobiology and Epigenetics, Freiburg, Germany; 2 Bioinformatics Unit, Max Planck Institute of Immunobiology and Epigenetics, Freiburg, Germany; 3 Genedata AG, Margarethenstrasse 38, 4053 Basel, Switzerland; 4 Faculty of Medicine, Albert Ludwigs University, Freiburg, Germany; 5 Present Address: Center for Bioscience Research and Education, Utsunomiya University, Utsunomiya, Tochigi, 321–8505 Japan

**Keywords:** Immunology, Immunogenetics, Innate immune cells, Innate lymphoid cells

## Abstract

Unconventional T cells, such as innate natural killer T cells (iNKT) cells, are an important part of vertebrate immune defences. iNKT recognise glycolipids through a T cell receptor (TCR) that is composed of a semi-invariant TCR α chain, paired with a restricted set of TCR β chains. Here, we show that splicing of the cognate *Trav11-Traj18-Trac* pre-mRNA encoding the characteristic Vα14Jα18 variable region of this semi-invariant TCR depends on the presence of *Tnpo3*. The *Tnpo3* gene encodes a nuclear transporter of the β-karyopherin family whose cargo includes various splice regulators. The block of iNKT cell development in the absence of *Tnpo3* can be overcome by transgenic provision of a rearranged *Trav11-Traj18-Trac* cDNA, indicating that *Tnpo3* deficiency does not interfere with the development of iNKT cells per se. Our study thus identifies a role for Tnpo3 in regulating the splicing of the pre-mRNA encoding the cognate TCRα chain of iNKT cells.

## Abstract (web summary)

iNKT cells recognise glycolipids via their T cell receptors. Here the authors implicate tnpo3 in the regulation of splicing of pre-mRNA encoding cognate TCR α chain for iNKT cells.

## Introduction

In the vertebrate immune system, conventional CD4^+^ and CD8^+^ αβ T cells provide the host with specific immune response capabilities, including the provision of effective memory responses. These conventional αβ T cells express a vast repertoire of structurally diverse antigen receptors and are activated by peptide antigen presented by the highly polymorphic classical MHC class I and class II molecules. By contrast, other thymus-derived populations of αβ T cells, such as invariant natural killer cells (iNKT) and mucosa-associated invariant T cells (MAIT), exhibit a much more restricted diversity of their antigen receptors, and recognise antigens presented by non-polymorphic non-classical MHC-like molecules^1,2^. These unconventional T cells, also known as innate T cells, acquire a memory-like phenotype in the thymus, unlike their adaptive cousins, which acquire this feature after antigen encounter in the periphery^3^. Thus, innate T cells do not require prior antigen experience to be able to rapidly respond to the recognition of their cognate antigens^4,5^. This pre-established memory-like phenotype is instrumental in providing the host with first-line defence capabilities that also impact the ensuing adaptive responses^6^. Instead of peptide ligands recognised by conventional T cells, iNKT cells recognise endogenous and exogenous glycolipids presented by CD1d, a non-polymorphic MHC class I-like molecule^7–10^. Mouse iNKT cells do so by virtue of expressing a semi-invariant TCR, which is composed of an invariant TCR α chain—consisting of Vα14 variable and Jα18 joining elements—that pairs with a limited number of TCR β chains, utilising mostly *Trbv2*, *Trbv7*, and *Trbv*8.2 variable genes^11–15^. The innate-like function of iNKT cells is exemplified by their rapid secretion of IFNγ and IL-4 cytokines after engagement of their cognate TCR by the αGalCer agonist; indeed, binding to αGalCer-CD1d tetramers is now considered a defining feature of the iNKT lineage^16,17^. The presence of innate T cells is not unique to mammals, since equivalent lymphocyte populations have been described in amphibians^18^. This indicates that these cells constitute a core component of vertebrate adaptive immunity.

iNKT cells share a common developmental pathway with conventional T cells until the CD4/CD8 double-positive (DP) stage, from where their developmental trajectories depart. Whereas the TCR repertoire of conventional T cells is shaped by selection on self peptide-MHC complexes expressed on thymic epithelial cells, future iNKT cells are selected by interaction with neighbouring DP cells. The development of iNKT cells from a DP thymocyte precursor^19,20^ requires the expression^21^ of the canonical TCR and its interaction with antigen/CD1d complexes expressed by DP cells^22,23^. This interaction is aided by engagement of homophilic receptors of the SLAM family across the DP-DP synapse^24^. Engagement of the canonical TCR by antigen-loaded CD1d on neighbouring cells triggers a cascade of events (including expression of *Egr2* and subsequently of *Plzf*) that initiates iNKT cell development and differentiation into different subtypes; iNKT1, iNKT2, and iNKT17, each express canonical transcription factors that drive their fate^6,25,26^ and establish the unique transcriptional programmes that underlie their distinct functional capacities^27–29^. Thus, iNKT cell development follows an instructive mode, whereby the formation and expression of the semi-invariant TCR constitutes the essential first step.

Primary transcripts of most genes undergo processing to generate mature mRNAs that can be translated into functional proteins. The splicing of pre-mRNAs depends on trans-acting factors, collectively referred to as the spliceosome, and cis-acting sequence motifs in the primary RNA transcript that demarcate the beginnings and ends of introns. Primary transcripts arising from rearranged antigen receptor genes are no exception and also contain several intronic sequences that must be removed during the maturation process^30,31^. TCR variable region genes possess one intron, typically positioned between the sequences encoding the signal peptide, and the body of the variable segment. A second intron occurs between the end of the J element and the first exon of the constant region gene; in the TCR α locus, which spans several tens of thousands of base pairs, this intron is very large, whereas the additional introns separating the various coding exons of the constant region gene are again smaller.

Owing to the complex, and often tissue-specific nature of the pre-mRNA splicing process, a large number of auxiliary splice regulators are employed to act in concert with the core spliceosome machinery, which often regulate the splicing of their own pre-mRNA^32^. Many of these splice regulators are delivered to the nucleoplasm by dedicated transporters of the β-karyopherin family^33^. Transportin 3 (Tnpo3) is a member of this class of proteins^34,35^. Mutations in the human *TNPO3* gene are associated with dominant limb-girdle muscular dystrophy 1F^36,37^, attesting to a surprising degree of tissue-specific activity of such splice regulators. Defects in members of the β-karyopherin family likely are indirectly associated with tissue-specific phenotypes. For instance, TNPO3 is known to bind to the serine/arginine-rich splicing factor SRSF6 (ref. ^35^), whose overexpression causes epithelial hyperplasia in the skin^38^; TNPO3 also binds to cleavage factor polyribonucleotide kinase subunit 1, whose absence results in changes in tRNA biogenesis and hence neurodegenerative disease^39,40^. Other TNPO3 cargoes include DEAH-box helicase 8 and DEAD-box helicase 46, lack of which give rise to aberrations in haematopoiesis^41,42^ and innate immunity^43^. We have shown previously that loss-of-function mutations of *tnpo3* in zebrafish and the orthologous *Tnpo3* gene in mice perturb conventional T cell development^44^.

Here, we identify an unexpectedly specific role of Tnpo3 in the pre-mRNA splicing process of the canonical Vα14-Jα18-TCRα chain of iNKT cells; in the absence of functional Tnpo3, iNKT cells do not develop.

## Results

### Conditional deletion of mouse *Tnpo3* in thymocytes

In the present study of mouse *Tnpo3* deficiency, we used a floxed allele of *Tnpo3* and a *pLck:Cre* driver to conditionally eliminate the function of *Tnpo3* in the αβ lineage of T cells at the DN3 stage^44^ (Supplementary Fig. 1a, b). The predicted mutant protein consists of the first 291 amino acids and closely resembles the mutant form identified in zebrafish (consisting of the first 202 amino acids)^44^; critically, it lacks the region required for interaction with serine-arginine-rich cargo proteins^35^, which is required for the transport function.

Transcriptome analysis of *Tnpo3*-deficient thymocytes (Supplementary Data 1) revealed substantial aberrations of the global splicing pattern. Compared to wild-type cells, the transcriptome of *Tnpo3*-deficient CD4^+^ cells is distinguished by a larger number of skipped exons (Supplementary Fig. 1c). Overall, 2.9% of all exon skipping events tested were significantly affected. In more than 95% of the significant exon skipping events, the exon is skipped in mutant cells, whereas in ~4% of the events the exon is included at higher levels in the mutant cells (Supplementary Fig. 1c). These results indicate that the inclusion of certain exons into mature mRNAs is a regulated process and dependent on the presence of Tnpo3, as expected from its role in regulating the transport of splicing factors into the nucleus. Interestingly, aberrant splicing in *Tnpo3*-deficient CD4^+^ cells was significantly enriched for genes encoding proteins containing RNA recognition motifs (InterPro entry IPR000504 [FDR < 0.01]); among the 39 genes in this category, the products of 25 genes are known to be involved in regulating pre-mRNA splicing, including 8 members of the heterogeneous nuclear ribonucleoprotein (HNRNP) and 6 members of the serine/arginine-rich splicing factor (SRSF) families of proteins. HNRNP proteins inhibit splicing, whereas SRSF proteins promote splicing^32^. Importantly, SRSF and HNRNP proteins also act on their own transcripts; the known autoregulation affects the equilibrium of splicing factor levels and the overall splicing landscape^32^. Of note, a substantial fraction of the splice regulators (here 10/25) encodes proteins that are known cargoes of the TNPO3 transporter^35^ (Supplementary Table 1), explaining the pervasive effects on the splicing landscape. Other examples of genes affected in both species include *Clk2*, encoding a serine/threonine and tyrosine dual specificity protein kinase, which regulates intranuclear localisation of serine-arginine-rich proteins and hence pre-mRNA splicing^45^, and of genes known to be important in lymphoid development such as Ikaros (*Ikzf1*)^46^ and protein tyrosine phosphatase receptor type C (*Ptprc*), an important regulator of haematopoietic differentiation^47^.

**Fig. 1:**
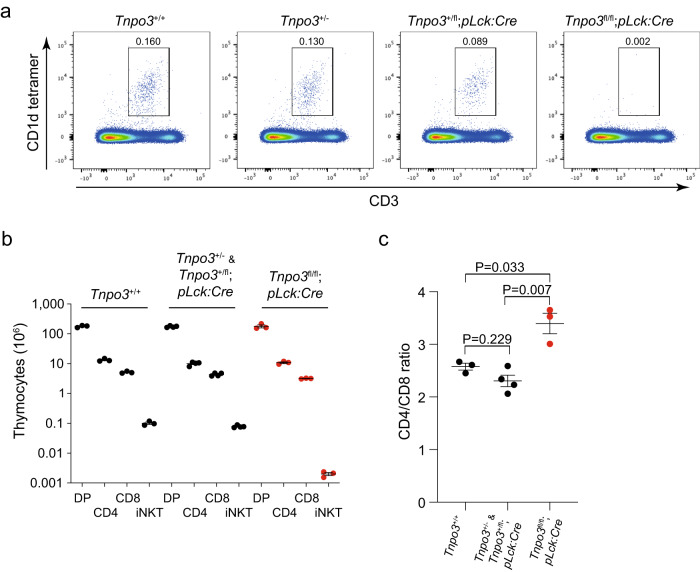
Lack of iNKT cells in the thymus of mutant *Tnpo3*^fl/fl^; *pLck:Cre* mice. **a** Flow cytometric profiles of total thymocytes isolated from the animals of the indicated genotypes stained with anti-CD3 antibodies and an αGalCer-CD1d tetramer. The profiles are representative of three animals each; the percentage of iNKT cells is indicated above the indicated area. **b** Differential effect of *Tnpo3* inactivation on thymocyte numbers. Note the lack of iNKT cells in the mutant animals; *n* = 3 biological replicates for all cell types. **c** The increased CD4/CD8 cell ratio in mutant animals reflects the more severely affected CD8 compartment in the mutants; *n* = 3 biological replicates for *Tnpo3*^+/+^ and *Tnpo3*^fl/fl^;*pLck:Cre*; *n* = 4 biological replicates for *Tnpo3*^+/-^/*Tnpo3*^+/fl^;*pLck:Cre*. **b,** **c**, t-test, two-tailed; mean ± s.e.m. are shown. Source data are provided as a Source Data file.

In the course of further investigating the T cell phenotype of conditionally *Tnpo3*-deficient mice, we found that these mice essentially lack type I NKT (iNKT) cells, defined as αGalCer-CD1d tetramer positive cells^16,17^, both in the thymus (Fig. 1a, b) and the periphery (Supplementary Fig. 2a, b); of note, CD8 single-positive (CD8) thymocytes are more affected by lack of *Tnpo3* than CD4 single-positive (CD4) thymocytes, resulting in a shifted CD4/CD8 ratio (Fig. 1c; Supplementary Fig. 2c). Collectively, these results suggest that the conditional ablation of *Tnpo3* at the DN2/3 stage of thymocyte development (Supplementary Fig. 1a, b) leads to the total lack of iNKT cells, reductions of thymocyte numbers and peripheral T cell lymphopenia. In order to confirm the conclusion that iNKT cells are absent in *Tnpo3*-deficient mice, we examined the response of wild-type and mutant mice to stimulation with αGalCer, an exquisitely selective agonist ligand for the iNKT-specific TCR^48^. After 2.5 h, wild-type mice respond to αGalCer injection by massive production of IL-4; this response was abolished in *Tnpo3*-deficient mice (Supplementary Fig. 3a). Likewise, IFN-γ production, measured 24 h after stimulation, was undetectable in mutant mice (Supplementary Fig. 3b). Thus, the lack of the Tnpo3 transporter in immature thymocytes initiates a cascade of events that ultimately results in an iNKT lineage-specific defect.

### Rescue of iNKT development

**Fig. 2:**
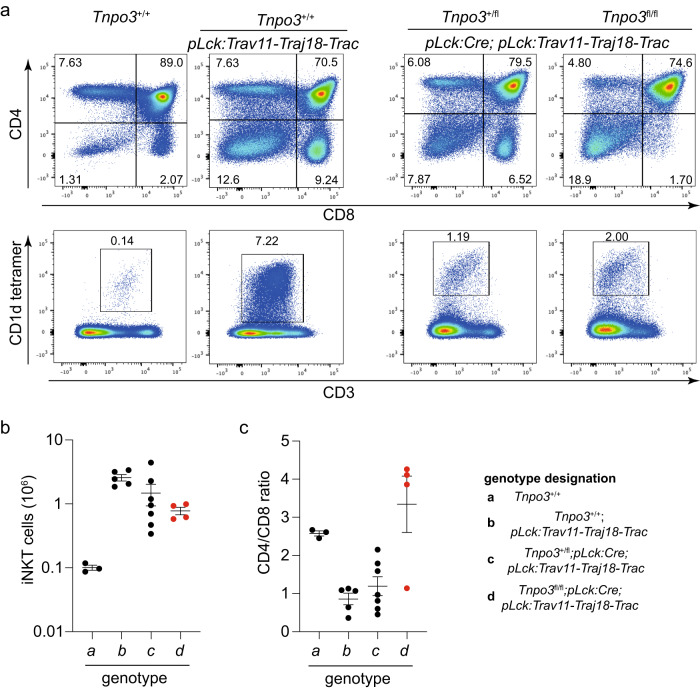
Restoration of iNKT development in *Trav11-Traj18-Trac* transgenic mice. **a** Flow cytometric profiles of total thymocytes isolated from the animals of the indicated genotypes stained with anti-CD4 and anti-CD8 antibodies (upper row) and anti-CD3 antibodies and an αGalCer-CD1d tetramer (bottom row). The profiles are representative of three animals each; the percentages of CD4, CD8, and iNKT cells are indicated. **b** Number of iNKT cells in mice of the indicated groups (genotype designation is indicated in a separate panel); *n* = 3 biological replicates for genotype a; *n* = 5 biological replicates for genotype b; *n* = 7 biological replicates for genotype c; *n* = 4 biological replicates for genotype d; mean ± s.e.m. are shown. **c** Variable CD4/CD8 cell ratios in mice of the indicated groups (genotype designation is indicated in a separate panel). The reduced CD4/CD8 ratio is indicative of precocious expression of the transgene; in the absence of *Tnpo3*, the reduced CD8^+^ thymocyte numbers skew the ratio in the opposite direction (c.f. Fig. 1c); *n* = 3 biological replicates for genotype a; *n* = 5 biological replicates for genotype b; *n* = 7 biological replicates for genotype c; *n* = 4 biological replicates for genotype d; mean ± s.e.m. are shown. Source data are provided as a Source Data file.

Splicing regulators are thought to be involved in specification and/or differentiation of individual cell types^49,50^; indeed, SRSF1 has been shown to be important for the differentiation of iNKT cells^51^. We hypothesised that the ectopic expression of the iNKT-lineage defining invariant Vα14-Jα18-TCRα chain in *Tnpo3*-deficient thymocytes would allow us to determine whether Tnpo3 is required for specification or differentiation of iNKT cells, or both. To this end, we generated transgenic mice expressing the Vα14-Jα18-TCRα chain under the control of the proximal *Lck* promoter (*pLck*). The activity of the *pLck* promotor is first detectable at the DN2/3 stage of thymocyte differentiation^52^, well before the CD4^+^/CD8^+^ double-positive (DP) stage, at which iNKT differentiation is initiated. If Tnpo3 were required not only for the initiation but also for the differentiation of iNKT cells, then their development would fail even after provision of the cognate receptor. Remarkably, however, enforced precocious expression of the *pLck:Trav11-Traj18-Trac* transgene resulted in the restoration of iNKT development in the thymus of mutant mice (Fig. 2a) and their presence in the peripheral lymphocyte compartment (Supplementary Fig. 4). Thus, the rescue of iNKT development by provision of the correctly assembled Vα14-Jα18-TCRα chain indicates that *Tnpo3* is not required for iNKT lineage specification per se, but rather important for the subsequent development of this lineage. Conversely, these observations suggest that the lack of the canonical invariant TCR α chain contributes to iNKT deficiency in *Tnpo3* mutants.

### Gene-specific splicing defect in the T cell receptor Vα locus

The expression of the semi-invariant iNKT TCR is a pre-requisite for agonist selection and subsequent initiation of iNKT lineage differentiation. One possible explanation for why iNKT development fails in mutant mice is that Tnpo3 is required for the provision of splice factors that regulate splicing of the *Trav11* gene, encoding the cognate Vα14 variable domain. In analogy to the situation in mice that genetically lack the *Traj18* element^13,14^ incompletely spliced *Trav11-Traj18-Trac* transcripts would abolish translation of the invariant TCRα chain and hence block the initiation of iNKT development.

**Fig. 3:**
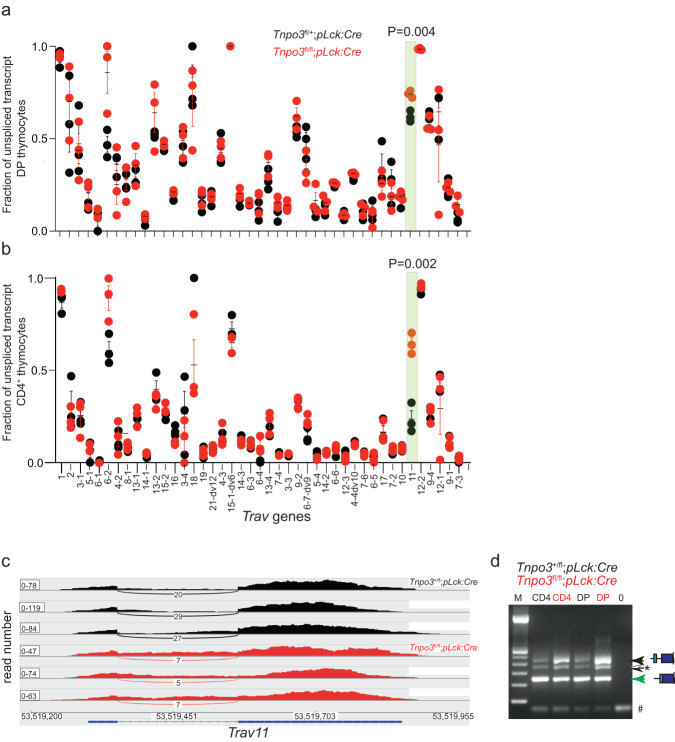
Aberrant splicing of mouse T cell receptor α variable (*Trav*) genes. Quantitative analysis of the fraction of unspliced transcripts of different *Trav* genes in *Tnpo3* heterozygous (*Tnpo3*^+/fl^; *pLck:Cre* [*n* = 3 biological replicates]) and homozygous (*Tnpo3*^fl/fl^; *pLck:Cre* [*n* = 3 biological replicates]) mutant DP (**a**) and CD4^+^ (**b**) thymocytes as determined by RNA-seq. The gene designations are indicated. Each data point represents the result of one mouse. A statistically significant difference (*P* < 0.01; beta-binomial test, two-sided, Bonferroni multiple testing correction) was observed in both populations only for *Trav11* (highlighted by green box with *P* values indicated); mean ± s.e.m. are shown. **c** Sashimi plot depicting the distribution of RNA sequencing reads across the *Trav11* gene in CD4^+^ thymocytes^,^ including those spanning splice donor and acceptor sites. The top three panels are derived from *Tnpo3* heterozygous (*Tnpo3*^+/fl^; *pLck:Cre*), the bottom three panels from homozygous (*Tnpo3*^fl/fl^; *pLck:Cre*) mutant mice. **d** Increased levels of unspliced *Trav11* transcripts in DP and CD4^+^ thymocytes isolated from *Tnpo3* heterozygous (*Tnpo3*^+/fl^; *pLck:Cre*) and homozygous (*Tnpo3*^fl/fl^; *pLck:Cre*) mutant mice as determined by standard RT-PCR; results are representative of three animals. The positions of the unspliced and spliced cDNAs of *Trav11* are indicated by the schematics to the right, as is the position of a heteroduplex (*) formed between the two forms of transcripts; the size of the spliced *Trav11* cDNA is 239 bp, that of the unspliced form 453 bp. M, marker lane; 0, control reaction without template; the band marked as “heteroduplex” was identified as such by sequencing. The fragment at the bottom (#) represents primer dimers. Source data are provided as a Source Data file.

To examine the possibility that *Tnpo3* deficiency affects the proper removal of the intron in *Trav11* and possibly other variable region genes, we determined the fractions of unspliced transcripts of *Trav* and *Trbv* genes in mutant mice. The genes encoding the variable segments of antigen receptors each possess one intron, which must be removed during pre-mRNA processing to generate mature mRNAs. In DP and CD4 thymocytes examined here, the levels of unspliced *Trav* transcripts vary among the different variable region genes (Fig. 3a, b). In general, the levels of unspliced *Trav* transcripts are higher in DP thymocytes (Fig. 3a) than in CD4 cells (Fig. 3b). In the *Tnpo3*-deficient background, only one variable region gene exhibited increased levels of unspliced transcripts in both DP and CD4 thymocytes at a significance of *P* < 0.01, *Trav11* (Fig. 3a, b), whereas no such consistent aberrations are observed for *Trbv* genes in DP and CD4 thymocytes (Supplementary Fig. 5). However, *Trav11* splicing does not completely fail in the absence of *Tnpo3*, since some *Trav11* transcripts are correctly spliced as seen from Sashimi plots (Fig. 3c) and RT-PCR (Fig. 3d). Collectively, these results demonstrate a remarkable selectivity of splicing defects associated with *Tnpo3* deficiency, and suggest that *Tnpo3* contributes to (but is not absolutely required for) the proper splicing of the *Trav11* variable region gene that encodes the Vα14 protein segment.

### *Tnpo3*-dependent activity of a *Trav11* intron-containing transgene

**Fig. 4:**
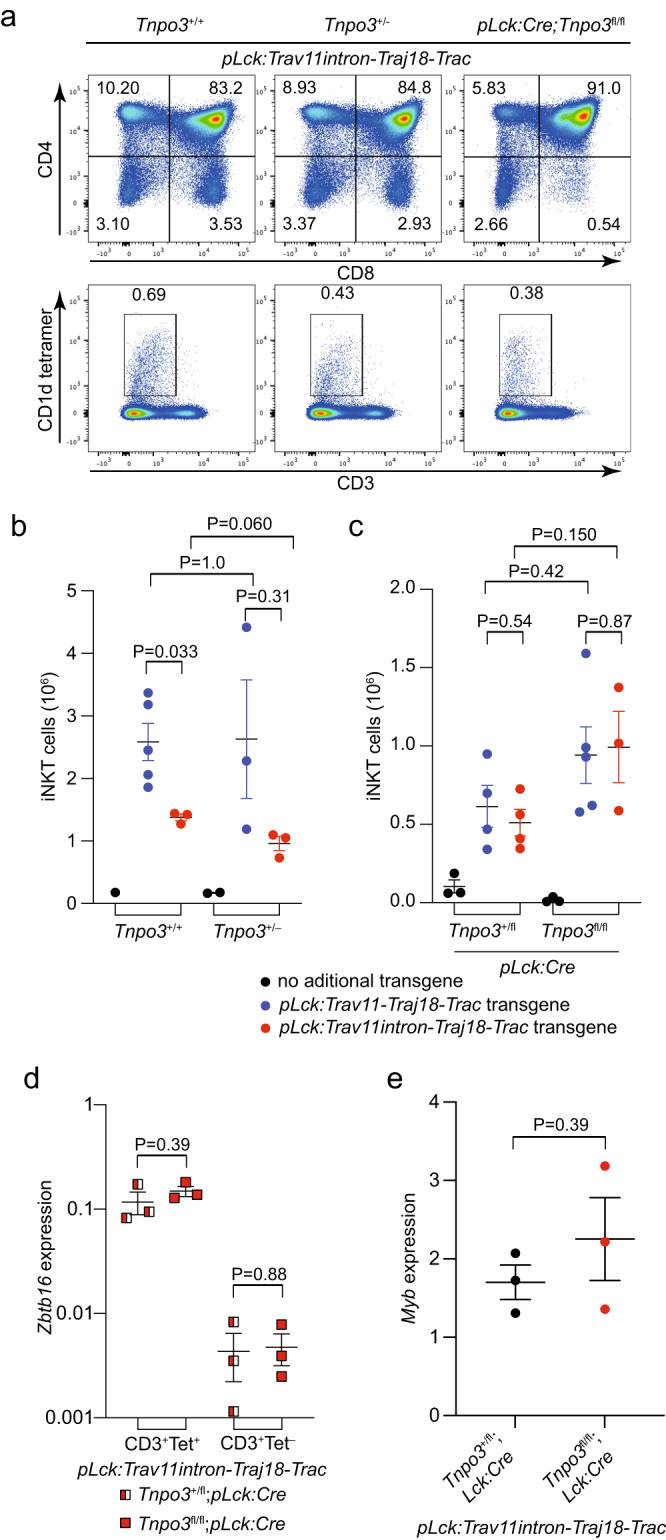
Restoration of iNKT development in *Trav11intron-Traj18-Trac* transgenic mice. **a** Flow cytometric profiles of total thymocytes isolated from the animals of the indicated genotypes stained with anti-CD4 and anti-CD8 antibodies (upper row) and anti-CD3 antibodies and an αGalCer-CD1d tetramer (bottom row). The profiles are representative of three animals each; the percentages of CD4, CD8, and iNKT cells are indicated. **b**, **c** Numbers of iNKT cells in the thymus of mice of the indicated genotypes. In (**b**), *n* = 1 for *Tnpo3*^+/+^; *n* = 5 biological replicates for *Tnpo3*^+/+^;*pLck:Trav11-Traj18-Trac*; *n* = 3 biological replicates for *Tnpo3*^+/+^;*pLck:Trav11intron-Traj18-Trac*; *n* = 2 biological replicates for *Tnpo3*^+/–^; *n* = 3 biological replicates for *Tnpo3*^+/–^;*pLck:Trav11-Traj18-Trac*; *n* = 3 biological replicates for *Tnpo3*^+/–^;*pLck:Trav11intron-Traj18-Trac*. In (**c**), *n* = 3 biological replicates for *Tnpo3*^+/fl^;*pLck:Cre*; *n* = 4 biological replicates for *Tnpo3*^+/fl^;*pLck:Cre*;*pLck:Trav11-Traj18-Trac*; *n* = 4 biological replicates for *Tnpo3*^+/fl^; *pLck:Cre*;*pLck:Trav11intron-Traj18-Trac*; *n* = 3 biological replicates for *Tnpo3*^fl/fl^;*pLck:Cre*; *n* = 5 biological replicates for *Tnpo3*^fl/fl^;*pLck:Cre*;*pLck:Trav11-Traj18-Trac*; *n* = 3 biological replicates for *Tnpo3*^fl/fl^; *pLck:Cre*;*pLck:Trav11intron-Traj18-Trac*. **d**, **e** Expression levels of *Zbtb16* and *Myb* genes (relative to *Hprt*) in the indicated cell populations of mice with the indicated genotypes as determined by qPCR; *n* = 3 biological replicates for all groups. **b**–**e**, t-test, two-tailed; mean ± s.e.m. are shown. Source data are provided as a Source Data file.

In order to address the role of Tnpo3 in *Trav11* splicing more directly, we generated a second mouse line that is transgenic for a construct in which the intron of *Trav11* is retained, designated *pLck:Trav11intron-Traj18-Trac*. To this end, we first compared the functional activities of the fully spliced *pLck:Trav11-Traj18-Trac* and the partially spliced *pLck:Trav11intron-Traj18-Trac* transgenes in supporting the generation of iNKT cells. Similar to the situation of *Tnpo3*^fl/fl^*; pLck:Cre;pLck:Trav11-Traj18-Trac* deficient mice (Fig. 2), iNKT development is also rescued in *Tnpo3*^fl/fl^*; pLck:Cre;pLck:Trav11intron-Traj18-Trac* mice (Fig. 4a). Interestingly, whereas the number of iNKT cells is identical for the intron-less construct when comparing *Tnpo3*^+/+^ and *Tnpo3*^+/–^ genotypes (*P* = 1.0), we observed lower numbers of iNKT cells in *Tnpo3*^+/–^ mice for the intron-containing construct (*P* = 0.060) (Fig. 4b). This observation suggests that possible effects of *Tnpo3* haploinsufficiency on *Trav11* splicing only become obvious when additional demands are generated by the presence of the intron-containing transgene. These observations hint at a rate-limiting function of Tnpo3 for the removal of the intron in the *Trav11* gene. Next, we examined the activities of the two transgenic constructs in *Tnpo3*-deficient mice. No difference was observed between the two constructs (Fig. 4c), reinforcing the notion that factor(s) other than Tnpo3 also contribute to *Trav11* splicing.

### Lack of endogenous *Trav11*-containing transcripts in *Tnpo3*-deficient thymocytes

In *Tnpo3*-deficient thymocytes, iNKT development completely fails (Fig. 1). However, the results of RNA-seq (Fig. 3) and transgenic rescue experiments (Fig. 4) clearly indicate that impaired *Trav11* splicing alone cannot account for this dramatic phenotype. Since no iNKT cells are present in *Tnpo3*-deficient mice, we set out to examine the presence and splicing pattern of endogenous *Trav11-*containing transcripts in the iNKT cells that develop in the mice additionally expressing the intron-containing construct. The rescued iNKT cells express normal levels of the *Zbtb16* gene, encoding a transcription factor whose expression is induced after formation of the cognate Va14-Ja18-TCR α chain^11,12^, and *Myb*, encoding a transcription factor important for iNKT differentiation^51^ (Fig. 4d, e).

**Fig. 5:**
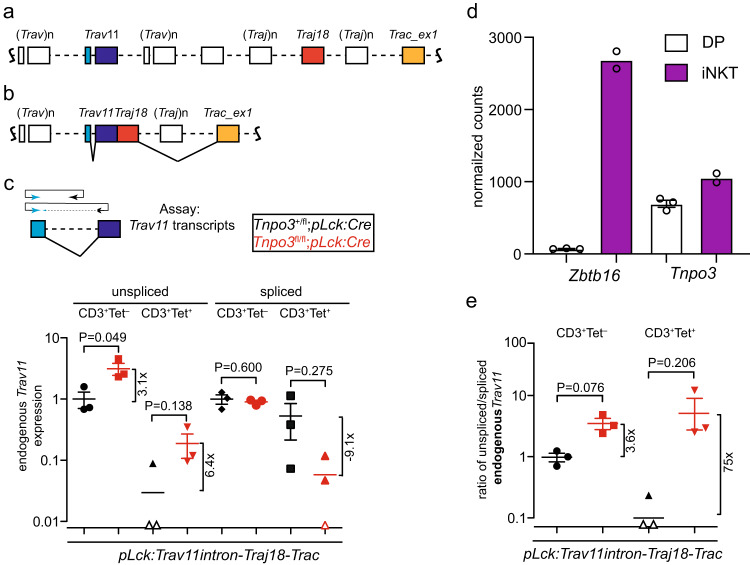
Lack of endogenous spliced *Trav11-Traj18-Trac* transcripts in *Tnpo3*-deficient thymocytes. **a** Schematic of the TCR α locus, highlighting the tandem arrays of *Trav* and *Traj* genes; note that the *Tcrd* locus, which is sandwiched between *Trav* and *Traj* genes is not shown for simplicity. **b** The recombined VJ genes are transcribed into a long primary transcript that undergoes two splicing steps, removing the *Trav* intron and the intron separating *Traj* and exon 1 of the *Trac* gene. **c** qPCR assay for the detection of endogenous *Trav11* splicing relative to *Gapdh* expression. The forward primer (blue) is located in the 5´-UTR of *Trav11*; the reverse primer is located either in the intron of *Trav11* (top scheme), or at the junction of the two *Trav11* exons and thus detects only the spliced *Trav11* transcript (bottom scheme). The colour code for the two genotypes is indicated. Tet^–^ and Tet^+^ refers to cells negative or positive in αGalCer-CD1d tetramer staining. *n* = 3 biological replicates for all conditions; mean ± s.e.m. are shown. **d** Expression levels of *Zbtb16* and *Tnpo3* genes in CD4^+^CD8^+^ double positive (DP) thymocytes and thymic iNKT cells (data taken from GSE37448); *n* = 3 biological replicates for DP cells; *n* = 2 biological replicates for iNKT cells; mean ± s.e.m. shown for DP cells; mean for iNKT cells. **e** Ratios of unspliced and spliced endogenous *Trav11* transcripts; data are from (**c**); *n* = 3 biological replicates for all conditions; mean ± s.e.m. are shown. Open data points in (**c**) and (**e**) indicate no signal; **c**, **e**, t-test, two-tailed. Source data are provided as a Source Data file.

The forward primer of the relevant qPCR assay targets the 5´-untranslated region of *Trav11*; reverse primers are situated either in the intron or at the junction of the first and second exons of the *Trav11* gene; in this way, we captured the unspliced and spliced versions of the *Trav11* variable gene (Fig. 5a–c). We examined these two transcript isoforms in purified populations of CD3^+^/αGalCer-CD1d^–^ and CD3^+^/αGalCer-CD1d^+^ (iNKT) thymocytes, respectively. Two genotypes were compared, which differ only by the presence or absence of *Tnpo3* activity; cells from *Tnpo3*^+/fl^;*pLck:Cre*;*pLck:Trav11intron-Traj18-Trac* transgenic mice served as controls, whereas cells from *Tnpo3*^fl/fl^;*pLck:Cre*;*pLck:Trav11intron-Traj18-Trac* transgenic mice constituted the experimental group.

Fully spliced endogenous *Trav11-*containing transcripts were found in the CD3^+^/αGalCer-CD1d^–^ populations of both control and *Tnpo3*-deficient cells (Fig. 5c). However, in purified CD3^+^/αGalCer-CD1d^+^ (iNKT) cells, endogenous *Trav11*-containing transcripts were robustly detectable only in the *Tnpo3*^+/–^ background; very small amounts, if any, were found in the iNKT cells that develop as a result of transgene expression in *Tnpo3*-deficient thymocytes (Fig. 5c). This finding supports the notion that spliced endogenous *Trav11-*containing transcripts are essentially below detection level in *Tnpo3*-deficient iNKT cells, indicating a particular requirement of Tnpo3 for iNKT cell function. Indeed, in wild-type mice, *Tnpo3* expression levels are higher in iNKT cells than in the DP cells (GSE37448; Fig. 5d).

In the *Tnpo3*-deficient background, the ratio of unspliced versus spliced endogenous *Trav11-*containing transcripts is drastically increased in CD3^+^/αGalCer-CD1d^+^ (iNKT) lineage cells (about 75-fold), whereas it is only moderately elevated in CD3^+^/αGalCer-CD1d^–^ cells (Fig. 5e). Note that the levels of unspliced *Trav11*-containing transcripts in *Tnpo3*-sufficient cells, and of spliced *Trav11*-containing transcripts in *Tnpo3*-deficient cells are close to the detection limit of the qPCR assay; this complication, and the possibility that unspliced/partially spliced *Trav11*-containing transcripts may be subject to nonsense-mediated decay both suggest that our results likely underestimate the magnitude of the changes reported above. Taken together, the qPCR results indicate that *Tnpo3*-deficiency disrupts the splicing of endogenous *Trav11-Traj18-Trac* transcripts and thus effectively prevents the development of iNKT cells. Transgenic *Trav11*-containing transcripts, which can be distinguished from endogenous transcripts by the use of an upstream primer binding to *pLck* promoter sequences (Supplementary Fig. 6a) were found in both CD3^+^/αGalCer-CD1d^–^ and CD3^+^/αGalCer-CD1d^+^ (iNKT) cells irrespective of *Tnpo3* genotype. Spliced isoforms of the transgenic transcripts are found in comparable levels in *Tnpo3*-heterozygous and *Tnpo3*-deficient thymocytes, whereas the levels of unspliced isoforms are modestly increased in *Tnpo3*-deficient cells (Supplementary Fig. 6b, c).

### Mechanistic basis of impaired *Trav11* splicing

**Fig. 6:**
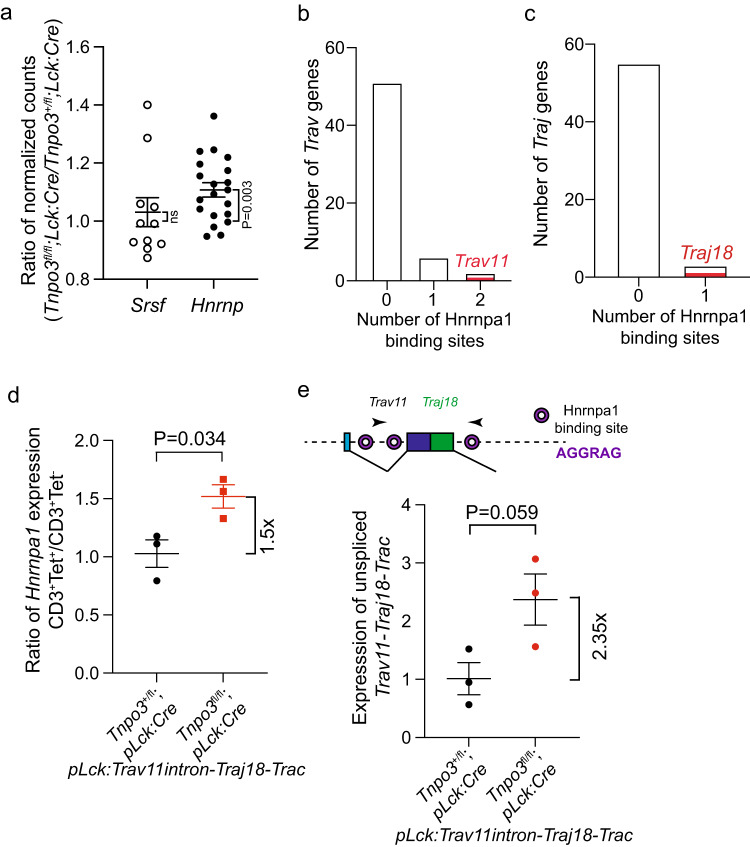
Mechanism of impaired splicing of *Trav11-Traj18-Trac* transcripts in *Tnpo3*-deficient thymocytes. **a** Ratio of expression levels of *Srsf* and *Hnrnp* gene family members in DP thymocytes of the two indicated genotypes; each dot represents a single gene; *n* = 11 *Srsf* genes; *n* = 19 *Hnrnp* genes. Single sample t-test, two-tailed; hypothetical mean = 1 to indicate no change between the two genotypes. The expression levels of *Srsf* and *Hnrnp* gene family members in DP thymocytes of the two genotypes are individually depicted in Supplementary Fig. 5e. Numbers of Hnrnpa1 binding sites in the introns of *Trav* genes (**b**) and downstream of *Traj* genes (**c**). Only two *Trav* genes possess two Hnrnpa1 binding sites, one of which is *Trav11* (red); only three *Traj* genes posses an Hnrnpa1 binding site downstream of the splice donor site, one of which is *Traj18* (red). The expression levels of these genes are depicted in Supplementary Fig. 5d. **d** Increased expression of *Hnrnpa1* in *Tnpo3*-deficient thymocytes as determined by qPCR; the ratios of expression levels in iNKT cells and CD3^+^ thymocytes are shown; *n* = 3 biological replicates for both conditions; mean ± s.e.m. are shown. **e** Expression levels of unspliced endogenous *Trav11-Traj18-Trac* transcripts in DP thymocytes of the indicated genotypes as determined by qPCR; *n* = 3 biological replicates for both conditions; mean±s.e.m. are shown. The locations of Hnrnpa1 binding sites in the transcript is schematically indicated at the top. **a,** **d,** **e**, t-test, two-tailed. Source data are provided as a Source Data file.

Next, we sought to establish a mechanism explaining the selective impairment of *Trav11* splicing. We began by considering the expression levels of genes encoding *Srsf* splice-promoting and *Hnrnp* splice-inhibiting genes to determine whether the transporter function of Tnpo3 perturbs the transcriptional landscape of these splice regulators. Because of substantially different expression levels, we compared the ratios of expression levels of individual genes of these gene families between DP thymocytes of *Tnpo3*^+/fl^;*pLck:Cre* and *Tnpo3*^fl/fl^;*pLck:Cre* mice, since iNKT cells do not develop in *Tnpo3*^fl/fl^;*pLck:Cre* mice. Despite considerable variability among individual family members, when considered as groups, no change was observed for *Srsf* genes (*P* = 0.55; single sample t-test, two-tailed), whereas higher levels of *Hnrnp* genes in *Tnpo3*-deficient thymocytes was observed (*P* = 0.0003; single sample t-test, two-tailed) (Fig. 6a). This prompted us to examine the possibility that the binding sites of inhibitory Hnrnp proteins might be differentially distributed among intronic sequences of *Trav* and *Traj* genes. We found that the *Trav11* intron contained 2 binding sites for Hnrnpa1 (ref. ^53^), and immediately downstream of *Traj18*, one binding site was found; by contrast, the majority of intronic sequences of *Trav* (Fig. 6b) and *Traj* (Fig. 6c) genes lacked such binding sites. This finding strongly suggested a potential role of the splice inhibitor Hnrnpa1 as a regulator of splicing of the *Trav11-Traj18-Trac* primary transcript. Thus, we would expect higher levels of *Hnrnpa1* transcripts as a result of *Tnpo3* deficiency specifically in mutant iNKT cells. This was indeed the case (Fig. 6d), suggesting the presence of a splicing landscape in *Tnpo3*-deficient iNKT cells that is unfavourable to proper splicing of *Trav11*-containing pre-mRNA transcripts. From this finding, we predicted that fully unspliced *Trav11-Traj18-Trac* transcripts should be present at higher levels in *Tnpo3*-deficient DP thymocytes. This was indeed the case (Fig. 6e), providing a mechanistic explanation for the minimal levels of properly spliced cognate *Trav11-Traj18-Trac* transcripts in *Tnpo3*-deficient thymocytes. Collectively, the selective absence of endogenous *Trav11*-containing transcripts confirms the susceptibility of iNKT lineage cells to the lack of *Tnpo3*, and indicates that the removal of the intronic sequence separating the two exons of *Trav11* and that separating *Traj18* and exon 1 of *Trac* are both impaired, resulting in negligible levels of cognate *Trav11-Traj18-Trac* transcripts in *Tnpo3* mutants.

## Discussion

TNPO3 is a member of a larger family of nuclear import receptors, which bind their specific cargo molecules and shuttle them across the nuclear pore complex^33^. The majority of cellular TNPO3 binding partners contain serine-arginine-rich domains^34,35^; these nuclear proteins possess RNA recognition motifs and play essential roles in pre-mRNA processing. Yet, the splicing of pre-mRNAs encoding Hnrnp proteins, which antagonise intron removal, and have so far not yet been identified as cargoes of human TNPO3, is also perturbed in *Tnpo3*-deficient cells. Therefore, it is reasonable to assume that any defects in pre-mRNA processing in cells lacking the Tnpo3 transporter are an indirect consequence of *Tnpo3* deficiency. Such indirect effects also offer an explanation for why the functional outcomes of *Tnpo3* deficiency may differ between tissues, for example by different degrees of functional redundancy with other members of the β-karyopherin family, and/or differential expression of TNPO3´s cargo proteins. Here, we demonstrate an unexpectedly specific effect of *Tnpo3* deficiency on the splicing of the *Trav11-Traj18-Trac* transcript encoding the canonical invariant TCRα chain of iNKT. Thus, without Tnpo3 activity, the expression by DP thymocytes of the characteristic invariant TCRα chain fails, abolishing the ability of these precursors to recognise their cognate ligands, diverse lipid antigens bound to CD1d, that are presented by neighbouring DP cells. Hence, both VJ rearrangement and expression of the canonical α chain is essential for the development of iNKT cells.

Previous work has identified how the choice of V and J elements affects the composition of the TCR repertoire. For instance, the frequency with which certain Vα elements are found in the CD4^+^ and CD8^+^ fraction of mature thymocytes depends on the nature of their CDR1 and CDR2 sequences^54–56^. Another source of bias is introduced by differential activities of V gene promoter elements, which also dictate their usage for TCRα or TCRδ chains^57^. Our present study adds a third layer of control, namely the differential splicing of Vα genes, a hitherto unrecognised aspect of TCR biology.

Previous work has shown that the splicing regulator Srsf1 is required for the development of iNKT cells^51^. The results of this study, together with the present findings, identify important roles for properly balanced stimulatory and inhibitory splicing activities in the iNKT lineage. Whereas constrained activity of Hnrnpa1 (and possibly of other inhibitory factors) as described here is essential to promote unimpeded splicing of *Trav11*-containing pre-mRNAs, the activity of Srsf1 is required to establish an environment that is conducive to proper splicing of pre-mRNAs of key transcription factors, such as Myb^51^.

Our results provide a mechanistic explanation for the impaired splicing of *Trav11-Traj18-Trac* transcripts. The presence of Hnrnpa1 binding sites in intronic sequences of this primary transcript, and the elevated levels of *Hnrnpa1* suggest that *Tnpo3* deficiency causes an inhibitory environment that blocks splicing of the cognate pre-mRNA, which may then undergo nonsense-mediated decay^58^. Although our results assign an important role to Hnrnpa1 in the regulation of *Trav11-Traj18-Trac* pre-mRNA splicing, other splice regulators likely participate in this process. However, under conditions of *Tnpo3* deficiency, their activity may be insufficient to generate high enough levels of mature *Trav11-Traj18-Trac* mRNA. In case of provision of supraphysiological levels of partially spliced *Trav11-Traj18-Trac* by transgenic over-expression, the threshold of mature mRNA levels is reached to support iNKT development.

Despite their overall sequence similarity, individual *Trav* genes can be distinguished by the presence or absence of binding sites for splicing regulators. Our finding of a seemingly complex regulation of *Trav* and *Traj* splicing (and possibly also those of other variable and joining genes) suggest that a comprehensive analysis of splicing regulation may uncover additional examples of lineage-specific effects.

Since TNPO3 has multiple cargo proteins, it is possible that *Tnpo3* deficiency would have detrimental effects also on the differentiation process of iNKT cells (for instance affecting the splicing of *Ikzf1* and *Ptprc* genes), in line with the intricate nature of the splicing code^59–61^ and the complex manner of pre-mRNA splicing regulation observed in the haematopoietic system^62^. Nonetheless, it is remarkable that the development (rather than differentiation) of an entire sub-lineage of T cells is absolutely dependent on Tnpo3; to the best of our knowledge, such phenotype has not yet been described for any splice regulator. It will be interesting to examine whether a similarly specific splicing mode regulates the provision of the transcripts encoding the Vα19 and Jα33 elements of the MAIT associated invariant TCRα chain.

## Methods

### Mice

The generation of *Tnpo3* conditional knock-out mice was achieved as follows. The ES cell line EPD0318_3_G02 (genetic background: C57BL/6N Agouti(A/a); allele name: *Tnpo3*^tm1a(KOMP)Wtsi^) was obtained from the KOMP Repository and used to derive chimaeric mice using standard procedures. Chimaeras were crossed with mice constitutively expressing FLP recombinase (B6:SJL-Tg(ACTFLPe)9205Dym/J [Jackson Laboratory stock number 003800])^63^ to remove the *lacZ/neo* cassette which is flanked by FRT sites. As a result, the ES-derived allele of *Tnpo3* possesses an exon 7 flanked by loxP sites. Constitutive deletion of exon 7 was achieved by crossing heterozygous mice with mice with a universal Cre-deleter strain (B6.C-Tg(CMV-cre)1Cgn/J [Jackson Laboratory stock number 006054])^64^. *Tnpo3* deficiency is embryonic lethal. T cell-specific deletion was achieved by crosses with mice transgenic for an *pLck:Cre* construct^52^. Wild-type and floxed alleles of *Tnpo3* were amplified from genomic DNA using primers 5′-GGAATTCAGTGCTCTGTACC and 5´-TCCAGCTCGGGATCCAATGC (amplicon sizes: wild-type allele, 227 bp; floxed allele, 342 bp); the deleted allele was amplified using primers 5′-GGAATTCAGTGCTCTGTACC and 5′-CAATTCCTGAAGCCACCCTG (amplicon sizes: deleted allele, 266 bp; wild-type allele, 947 bp; floxed allele, 1072 bp). Partial *Tnpo3* cDNAs were amplified using primers 5′-CGAAGCTGCTTCAGACTGTG (located in exon 6) and 5′-ATGTTCTCCTAGTCGGTACC (located in exon 8); amplicon size of the wild-type form, 331 bp; of the mutant form, 192 bp. The mouse floxed *Tnpo3* allele^44^ was maintained on the C57BL/6J background. Mice expressing the various forms of the iNKT-specific TCR α chain gene (see below) were generated by injection of the linearised constructs into FVB pronuclei according to standard techniques. Mice of mixed B6/FVB background were analysed at 6 weeks of age without regard to sex as no sex-dependent variation of phenotypes was observed. All animal experiments were approved by the institute’s review committee and conducted under license from and approved by the local government (Regierungspräsidium Freiburg) (license 35-9185.81/G-15/35).

### Transgenic constructs

The *Trav11-Traj18-Trac* construct was assembled by standard cloning techniques and is composed of the following sequences: proximal *Lck* gene promotor (nucleotide 163,417 to nucleotide 159,952 in Genbank accession number AL606921.6); *Trav11* gene^65^ cDNA sequences (nucleotide 12 to nucleotide 834 in Genbank accession number M14506.1); human growth hormone 3′- sequences (nucleotide 2596 to nucleotide 4107 in Genbank accession number KR632635.1, directly followed by nucleotide 544 to nucleotide 1,169 in Genbank accession number KU665646.1). Likewise, the *Trav11intron-Traj18-Trac* construct is composed of the following sequences: proximal *Lck* gene promotor (nucleotide 163,417 to nucleotide 159,952 in Genbank accession number AL606921.6); *Trav11* gene cDNA sequences (nucleotide 12 to nucleotide 65 in Genbank accession number M14506.1, followed by nucleotide 396,943 to nucleotide 397,156 in Genbank accession number NG_0070441 [corresponding to the *Trav11* intron sequences], followed by nucleotide 66 to nucleotide 834 in Genbank accession number M14506.1); human growth hormone 3′- sequences (nucleotide 2596 to nucleotide 4107 in Genbank accession number KR632635.1, directly followed by nucleotide 544 to nucleotide 1169 in Genbank accession number KU665646.1). The copy numbers of the two transgenic lines expressing these constructs as determined by qPCR against a single-copy gene are as follows: *Trav11-Traj18-Trac*, 11 copies; *Trav11intron-Traj18-Trac*, 7 copies; copies per diploid genome.

### RNA extraction and cDNA synthesis

Total RNA was extracted using TRI Reagent (Sigma) following the manufacturer’s instructions. After treatment with Cloned DNaseI (Takara), RNA extraction using TRI Reagent was repeated. Superscript III Reverse Transcriptase (Invitrogen) and random hexamer primers were used for cDNA synthesis from total RNA.

### RT-PCR

RT-PCR was carried out as described previously^66^. Primer sequences can be found in Supplementary Table 2.

### qPCR

qPCR was carried out using SYBR Premix Ex Taq (Takara); alternatively, qPCR was performed using FastStart Universal Probe Master (ROX) mix (Roche) using 5′ FAM (6-carboxyfluorescein) labelled hydrolysis probes from either the Universal Probe Library (Roche), or custom synthesised (Eurofins Genomics) with 5′ FAM and 3′ TAMRA labels. The 7500 Fast system (Applied Biosystems) was used to detect the generated signals. Primer sequences can be found in Supplementary Table 2.

### Flow cytometry

Thymocyte and splenocyte suspensions were prepared by mechanical liberation, best achieved by gently pressing organs through 40 μm sieves; subsequent flow cytometric analyses were carried out using Fortessa instruments (Dako Cytomation-Beckman Coulter). For analysis of *Tnpo3* mutant mice, analytical flow cytometry^67^ was carried out using the following antibodies: FITC-conjugated anti-CD4 (clone GK1.5; BioLegend Cat# 100405; 1:1000 dilution); PE-conjugated anti-CD8α (clone 53-6.7; eBioscience Cat #12-0081-82; 1:800 dilution); PerCP-Cy5.5-conjugated anti-CD3ε (clone 145-2C11; Biolegend Cat# 100327; 1:100 dilution); APC-conjugated anti-CD19 (clone MB19-1; eBioscience Cat#17-0191-82; 1:100 dilution); PE-conjugated CD1d Tetramer (Proimmune Cat# D001-2X; 1:200 dilution); FITC-conjugated anti-TCRγδ (clone eBioGL3; eBioscience Cat#11-5711-82; 1:100 dilution); PE-conjugated anti-TCRβ (clone H57-597; eBioscience Cat#12-5961-82; 1:300 dilution). For preparative flow cytometry, the following antibodies were used: APC-Cy7-conjugated anti-CD4 (clone GK1.5; Biolegend Cat#100413; 1:400 dilution); FITC-conjugated anti-CD8a (clone 53-6.7; eBioscience Cat#11-0081-82; 1:800 dilution); PerCP-Cy5.5-conjugated anti-TCRβ (clone H57-597; eBioscience Cat#45-5961-82; 1:100 dilution); PE-Cy7-conjugated anti-CD24 (clone M1/69; eBioscience Cat#15-0242-82; 1:1000); PE-conjugated CD1d Tetramer (Proimmune Cat# D001-2X; 1:400 dilution).Gating strategy for live cells is exemplified in Supplementary Fig. 7.

Information on validation of antibodies can be found on the manufacturers’ websites (Biolegend: https://www.biolegend.com/en-us/antibodies-reagents; eBioscience: https://www.thermofisher.com/de/de/home/life-science/antibodies/ebioscience.html; Proimmune: https://www.proimmune.com/). BD FACSDiva software (up to version 6.1.3 was used for data collection; FloJo software (up to version 10.9.0) was used for data analysis; statistical analysis was performed with GraphPad Prism (up to version 9.5.1) software.

### Cytokine ELISA

Serum cytokine levels after α-galactosylceramide injection were measured essentially following the method of ref. ^14^; 1 mg of α-galactosylceramide (Abcam) in PBS was injected intravenously into control (*Tnpo3*^+/fl^; *pLck:Cre*) and mutant (*Tnpo3*^fl/fl^; *pLck:Cre*) mice. Blood serum was collected from the mice after 2.5 and 24 h. IFN-γ and IL-4 concentrations in the serum were measured using Quantikine ELISA (R&D Systems).

### RNA isolation and sequencing

Total RNA was extracted from purified mouse CD4^+^CD8^+^TCRβ^int^CD24^high^ (thereafter referred to as DP) and CD4^+^CD8^-^TCRβ^high^CD24^low^ (hereafter referred to as CD4^+^ single-positive) cells using RNeasy Mini kit (Qiagen), including a DNaseI treatment step. RNA quality was determined using the 2100 Bioanalyzer instrument (Agilent) and RNA concentration was determined using Qbit 2.0 Fluorometer (Life Technologies). To examine expression levels and splicing patterns in mouse cells, 300 ng of total RNAs per sample (three biological replicates each) were used for library preparation using the TruSeq Stranded Total RNA kit with Ribo-Zero Human/Mouse/Rat (RS-122-2203, Illumina). The libraries were sequenced on an Illumina HiSeq 2500 instrument in 75 bp paired-end mode using 0.5 lanes per sample.

### Computational analysis of mouse RNA-seq data

*(a) Alignment of RNA-seq reads*. The raw RNA-seq reads were mapped against the mouse genome assembly GRCm38 with the spliced sread aligner TopHat2 version 2.0.13 (ref. ^68^). Reads were first mapped to the transcriptome provided by the mouse gene annotation of GENCODE release M4 (ref. ^69^). Unmapped reads were subsequently aligned against the mouse genome. TopHat2 was run with the library type parameter “fr-secondstrand” as the RNA-seq library was prepared with a strand-specific protocol. Strand-specific library type, fragment length mean and standard deviation were determined on a subsample of 1 million reads with RSeQC version 2.4 (ref. ^70^), and provided to TopHat2. All other TopHat2 parameters were set to default. *(b) Analysis of differential gene expression and exon usage*. The numbers of sequenced fragments per annotated gene (based on GENCODE M4) were quantified with featureCounts version 1.4.5-p1 (ref. ^71^). Fragments were only counted if both ends aligned on the same chromosome with a minimum mapping quality of 10 and in agreement with the strand orientation of the annotated gene. Reads per exons were counted with HTSeq version 0.6.1 (ref. ^72^) using analogous parameters, but including only transcripts with GENCODE tag “basic”. Differential gene expression analysis was performed with the Bioconductor package edgeR version 3.12.0 (ref. ^73^). Weakly expressed genes with less than 0.2 counts-per-million (CPM) in at least three samples were filtered out. Dispersion estimation and testing for differential expression between heterozygous control (*Tnpo3*^+/fl^; *pLck:Cre*) and homozygous mutant (*Tnpo3*^fl/fl^; *pLck:Cre*) samples were performed using the quasi-likelihood (QL) method^74^, robustified against potential outlier genes. Exon read counts were normalised with the Bioconductor package DEXSeq version 1.16.0 (ref. ^75^). The exon expression scatter plot shows the mean read counts, normalised by sample-specific size factors, per exon for genotypes *Tnpo3* heterozygous (*Tnpo3*^+/fl^; *pLck:Cre*) vs. homozygous (*Tnpo3*^fl/fl^; *pLck:Cre*) mutant mice. The exon usage of alternative transcript isoforms is visualised by Sashimi plots^76^. *(c) Analysis of differential alternative splicing events*. The BAM files with TopHat2 aligned reads were filtered to include only reads that overlap with annotated transcripts in the correct strand orientation. The filtered BAM files for both genotypes were then used as input for the differential alternative splicing (AS) analysis with rMATS version 3.0.9 (ref. ^77^). Of all reads, rMATS only considers unique, properly paired reads that are mapped without insertions and deletions. The mean fragment size of the paired-end samples and its standard deviation were determined as described above and provided to rMATS. All other parameters were set to default. Based on the provided GENCODE M4 annotation, rMATS builds a database of possible AS events that are either (i) already annotated by alternative gene isoforms or (ii) novel and supported by reads covering un-annotated exon junctions between annotated exons within a gene. The reported AS events are of the following types: exon skipping, retained intron, mutually exclusive exons, alternative 5′ donor or 3′ acceptor splice sites. We selected for each type all AS events with at least 5% difference in the mean exon inclusion levels that were identified by reads spanning splice junctions at 1% FDR. Functional enrichment analysis of genes with significant AS events was performed with the DAVID Functional Annotation Clustering tool^78,79^. For each set of genes with significant AS events of a specific type (e.g. exon skipping), the genes were tested for functional enrichment by comparing them to the background of all genes that were tested for the respective AS event type. *(d) Quantification of Tcra and Tcrb transcript levels*. The abundance of spliced and unspliced transcripts was quantified with Salmon version 0.4.2 (ref. ^80^). The index included all spliced and unspliced *Tcra* and *Tcrb* transcripts based on GENCODE M4 annotation, excluding pseudogenes and *Trcra* gene duplicates (identified by trailing “d” or “n” in gene name). Salmon was called in extra-sensitive search mode and with 98% required read coverage. The beta-binomial test^81^ was used to test for significant differences in the splicing rate and the relative abundance of transcripts between genotypes; *P* values were adjusted for multiple testing by Bonferroni correction. RNA-seq data are deposited in NCBI´s Gene Expression Omnibus^82^ and are accessible through GEO accession number GSE77137.

### Reproducibility and statistics

The numbers of biological replicates are indicated in the relevant figure legends. No statistical method was used to predetermine sample size; no data were excluded from the analysis; the experiments were not randomised; investigators were not blinded to allocation during experiments and outcome assessment. Data were analysed and visualised using the Prism9 software suite. Statistical significance tests used are indicated in the figure legends and the methods section. Groups were deemed significantly different, when *P* < 0.05 or *P* < 0.01, as indicated. In graphs, each data point represents a biological replicate.

### Reporting summary

Further information on research design is available in the Nature Portfolio Reporting Summary linked to this article.

## Supplementary information

Supplementary Information

Description of Additional Supplementary Files

Supplementary Data 1

Reporting Summary

## Data Availability

All data needed to evaluate the conclusions in the paper are present in the paper or the Supplementary Materials. RNA-Seq data are deposited in NCBI´s Gene Expression Omnibus under GEO accession number GSE77137. The data in Fig. 5d were taken from publicly available sources (GSE37448). Source data are provided with this paper.

## Source data

Source Data
