# Supplementary material for: Tnpo3 controls splicing of the pre-mRNA encoding the canonical TCR α chain of iNKT cells
Source: Nat Commun. 2023 Jun 20;14:3645. doi: 10.1038/s41467-023-39422-4 (PMC10282035; doi:10.1038/s41467-023-39422-4)
Supplement: Supplementary file 1 — Supplementary Information [file 41467_2023_39422_MOESM1_ESM.pdf]

Supplementary Material
for

Tnpo3 controls splicing of the pre-mRNA encoding the canonical TCR α chain of iNKT cells

Norimasa Iwanami, Andreas S. Richter, Katarzyna Sikora, and Thomas Boehm

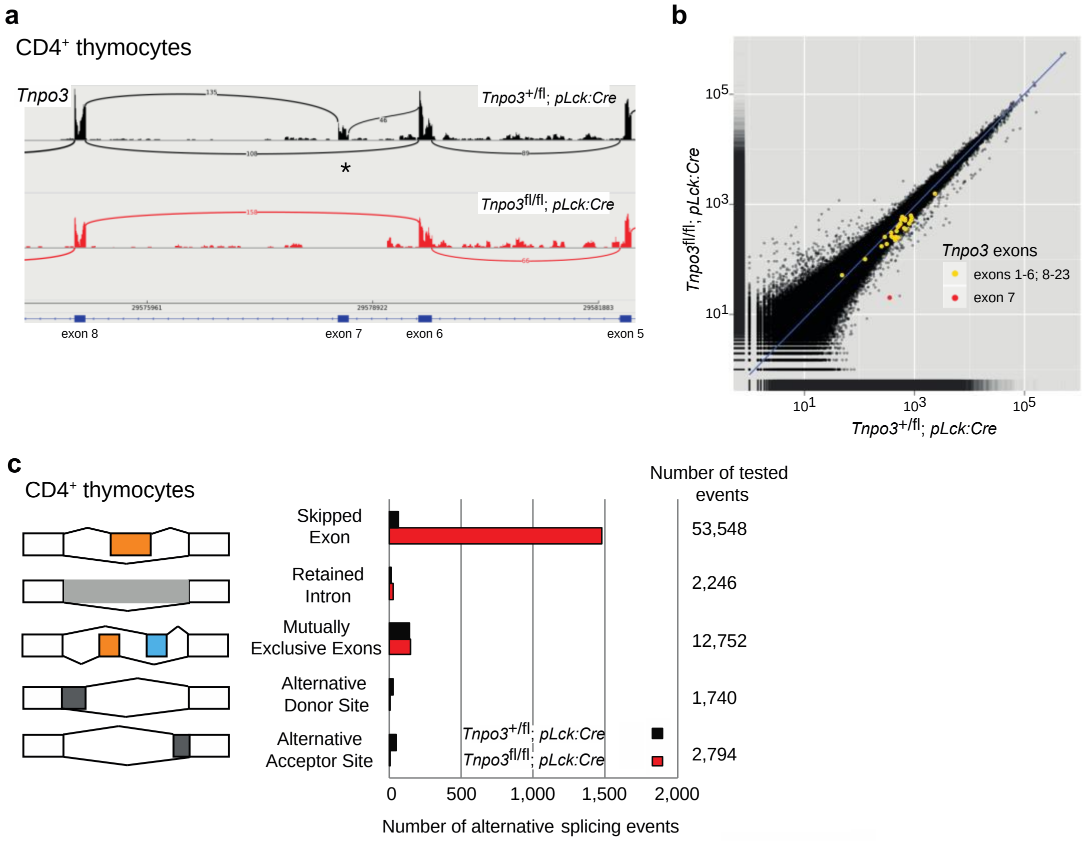

Supplementary Fig. 1 Characterization of the conditional *Tnpo3* knock-out allele. a Sashimi plot depicting the distribution of RNA sequencing reads across the *Tnpo3* gene in heterozygous (*Tnpo3*$^{+/fl}$; *pLck:Cre* [black histogram]) and homozygous (*Tnpo3*$^{fl/fl}$; *pLck:Cre* [red histogram]) mutant CD4^{+} thymocytes. Exon read coverage is shown as a histogram and reads overlapping exon/exon junctions are shown as arcs connecting exon borders; traces are representative of 3 biological replicas. The absence of mRNAs containing exon 7 (coordinates on chromosome 6: nucleotides 29,578,463 to 29,578,601) in the homozygous mutant cells indicates complete deletion of this exon in the genome of T cells. **b** Mean read counts per exon for heterozygous (*Tnpo3*$^{+/fl}$; *pLck:Cre*) and homozygous (*Tnpo3*$^{fl/fl}$; *pLck:Cre*) mutant DP and CD4^{+} thymocytes. Exons of the *Tnpo3* gene are highlighted in yellow; the conditionally deleted exon 7 of *Tnpo3* is highlighted in red. **c** Regulation of alternative splicing by *Tnpo3* in mouse CD4^{+} thymocytes. The bar chart shows the number of significant alternative splicing (AS) events (schematics are indicated on the left) in cells isolated from *Tnpo3* heterozygous (*Tnpo3*$^{+/fl}$; *pLck:Cre* [n=3 biological replicates]) and homozygous (*Tnpo3*$^{fl/fl}$; *pLck:Cre* [n=3 biological replicates]) mutant mice at a false discovery rate (FDR) of 1% for five different AS event types. The total numbers of possible AS events that were tested are indicated on the right. Homozygous mutant cells exhibit greater numbers of skipped exons.

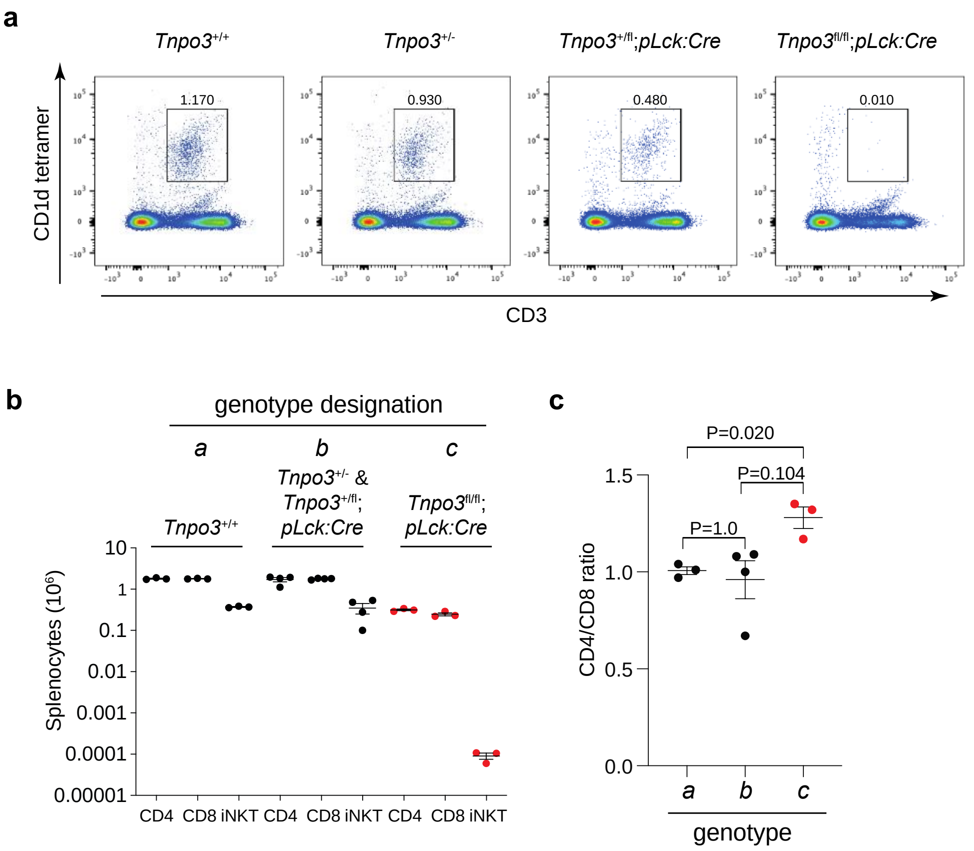

Supplementary Fig. 2 Lack of iNKT cells in the spleen of mutant *Tnpo3*$^{fl/fl}$; *Lck:Cre* mice. **a** Flow cytometric profiles of total splenocytes isolated from the animals of the indicated genotypes stained with anti-CD3 antibodies and an αGalCer-CD1d tetramer. The profiles are representative of 3 animals; the percentage of iNKT cells is indicated above the indicated area. **b** Differential effect of *Tnpo3* inactivation in peripheral T cells. Note the reduction of CD4$^+$ and CD8$^+$ single-positive T cells in the mutant animals, and the lack of iNKT cells in the mutant animals. **c** The increased CD4/CD8 cell ratio in mutant animals reflects the more severely affected CD8 compartment. For panels **b** and **c**, n=3 biological replicates for genotypes *a* and *c*; n=4 biological replicates for genotype *b*; mean±s.e.m. are shown; t-test, two-tailed.

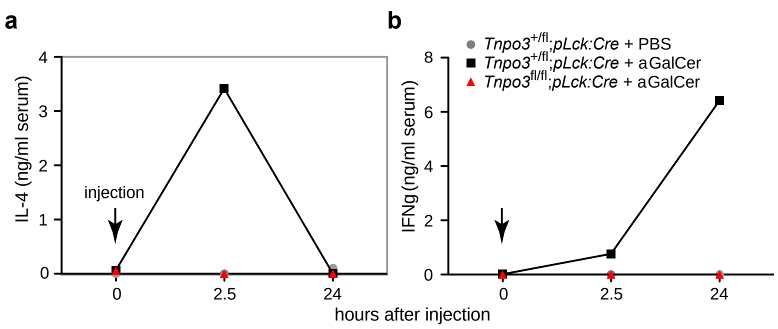

Supplementary Fig. 3 Lack of iNKT cells in *Tnpo3*-deficient mice. Cytokine response after injection of αGalCer. The early IL-4 (**a**) and the late IFNγ (**b**) responses are absent in *Tnpo3*-mutant mice. The genotypes and stimuli used are indicated; serum was taken at the indicated times points just before (t=0) injection (arrow) and 2.5 and 24 hours later. Data are representative of two independent experiments (n= 2 mice per genotype in each). Mean values are indicated. In control animals, serum concentrations were below detection levels. This experiment was repeated with another set of two mice in each category with similar results.

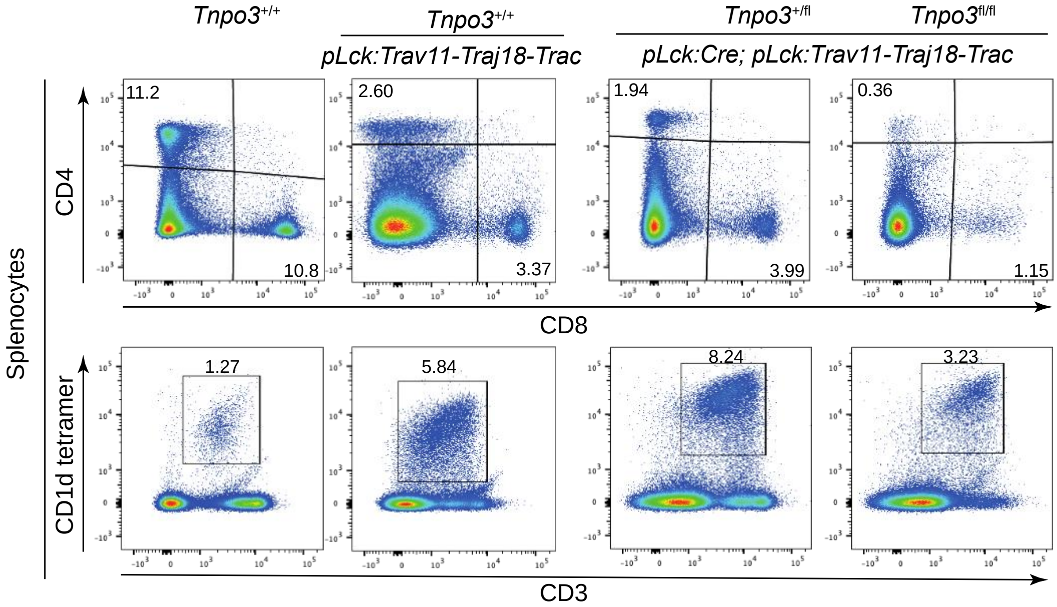

Supplementary Fig. 4 Restoration of iNKT development in *Trav11-Traj18-Trac* transgenic mice. Flow cytometric profiles of total splenocytes isolated from the animals of the indicated genotypes stained with anti-CD4 and anti-CD8 antibodies (upper row) and anti-CD3 antibodies and an αGalCer-CD1d tetramer (bottom row). The profiles are representative of 3 animals each; the percentages of CD4, CD8, and iNKT cells are indicated.

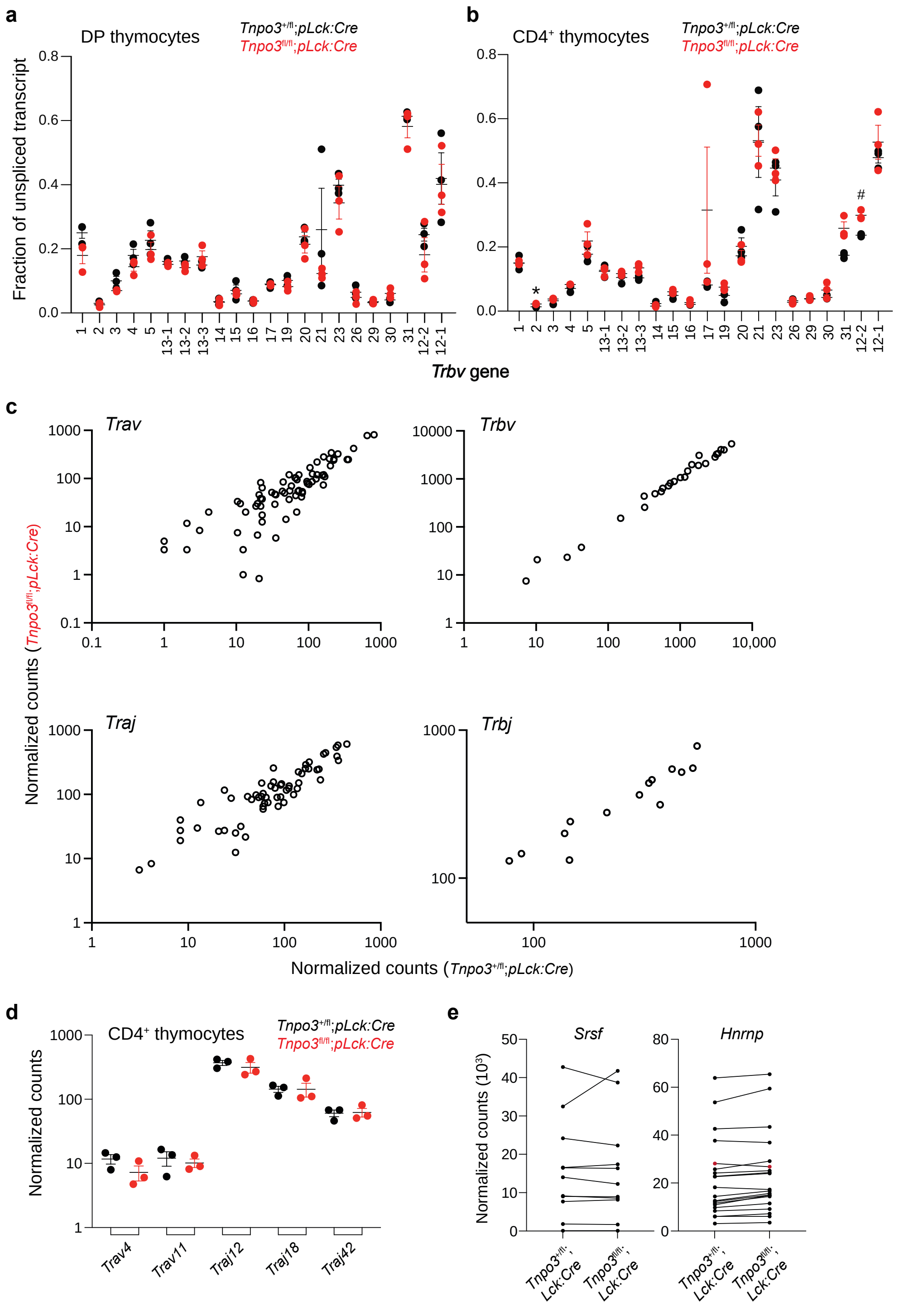

Supplementary Fig. 5 Aberrant splicing of mouse T cell receptor β variable (*Trbv*) genes. a and **b** Fractions of unspliced transcripts of different *Trbv* genes in *Tnpo3* heterozygous (*Tnpo3*$^{+/\mathrm{fl}}$; *pLck:Cre* [n=3 biological replicates]) and homozygous (*Tnpo3*$^{\mathrm{fl/fl}}$; *pLck:Cre* [n=3 biological replicates]) mutant DP (a) and CD4^{+} (b) thymocytes. The gene designations are indicated. Each data point represents the result of one mouse. Statistically significant differences (P≤0.01; beta-binomial, two-tailed; Bonferroni multiple testing correction) were not observed for any *Trbv* gene in DP thymocytes; in CD4^{+} thymocytes, statistically significant differences were observed for *Trbv2* and *Trbv12-2* (indicated by *

(P=0.0018) and # (P=0.0024), respectively); however, for *Trbv2*, the absolute level of unspliced transcripts was less than 3% and the magnitude of change was less than 2-fold; for *Trbv12-2* the magnitude of change was a mere 1.25-fold. These small changes are deemed biologically irrelevant. All other Trbv genes showed no statistically significant difference. **c** Usage of individual *Trav, Trbv, Traj,* and *Trbj* elements in the repertoire of DP thymocytes of the two indicated genotypes; each data point represents the mean value (n=3 biological replicates) for an individual gene. **d** Expression levels of *Trav* genes possessing two Hnrnpa1 binding sites in the intron; expression levels of *Traj* genes possessing one Hnrnpa1 binding site downstream of the splice donor sites (see Fig. 6a); n=3 biological replicates for all genes; mean±s.e.m. are shown. **e** Expression levels of *Srsf* (left panel) and *Hnrnp* (right panel) family member genes in DP thymocytes of the indicated genotypes; the Hnrnpa1 *gene* is indicated (red dots). Each data point represents the mean value (n=3 biological replicates) for an individual gene.

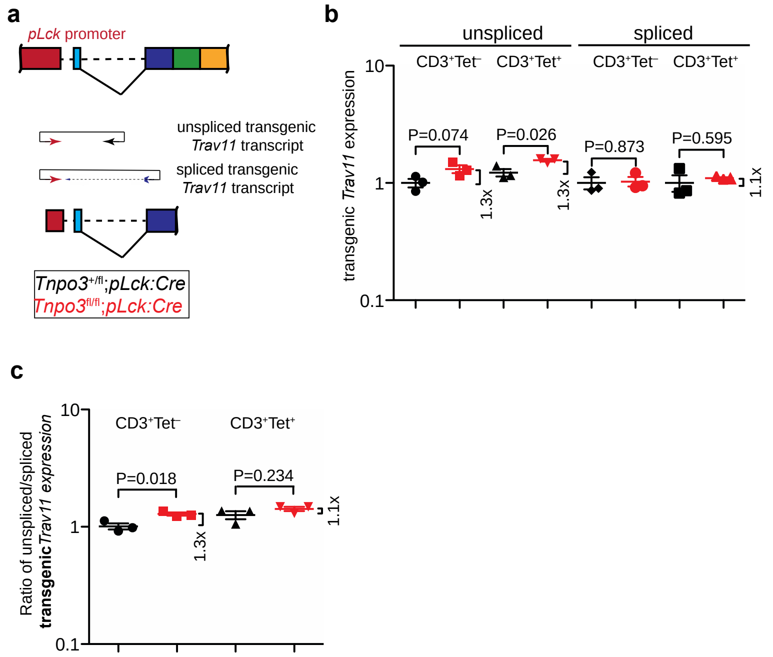

Supplementary Fig. 6 Expression of TCR alpha transcripts in *Tnpo3*-deficient thymocytes expressing a *Trav11-intron-Traj18-Trac* transgene. a Schematic of the qPCR assay to determine the splicing efficiency of the *Trav11* intron in the *Trav11*(intron)-*Traj18-Trac* transgene; the assay is analogous to that used in Fig. 5c, except that the forward primer is located in the *pLck* promoter sequence (red box). **b** Expression levels of unspliced and spliced transgenic *Trav11* transcripts in the indicated cell types isolated from mice of two *Tnpo3* genotypes as determined by qPCR. **c** Ratio of unspliced and spliced transgenic *Trav11* transcripts in the indicated cell types isolated from transgenic mice of two *Tnpo3* genotypes (right panel). For panels **b**, **c**, mean±s.e.m. of n=3 biological replicates are shown for each cell type and condition; t-test, two-tailed.

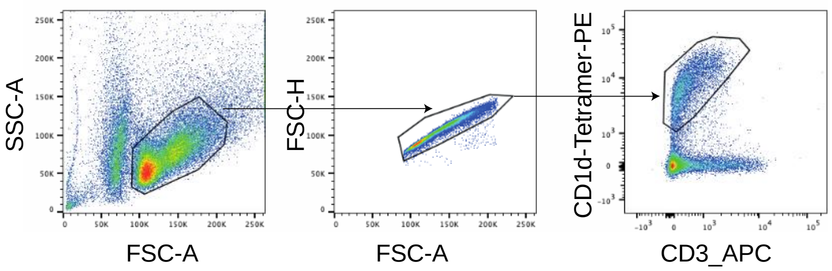

Supplementary Fig. 7 Gating scheme to analyse lymphocyte populations. Live cells were gated for desired forward and side scatter light characteristics (left panel), then gated for single cells (middle panel), and finally gated for $CD3^{+}$CD1d-tetramer^{+} cells (right panel).

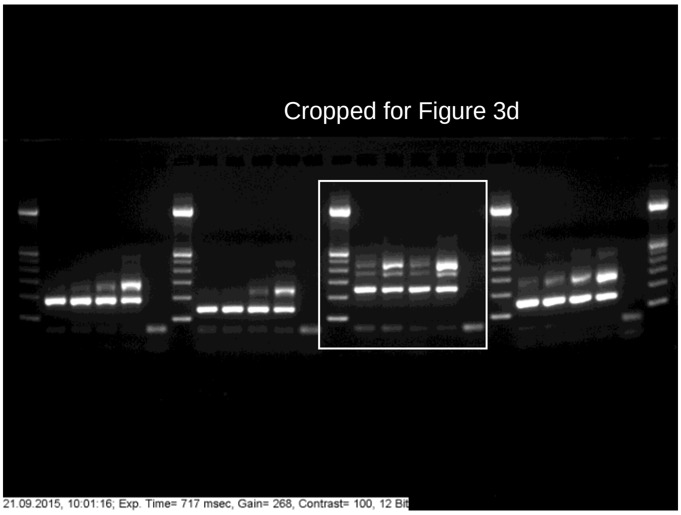

Supplementary Fig. 8 Uncropped gel picture for Fig. 3d. The relevant section is indicated.

Supplementary Table 1 Genes encoding proteins with RNA recognition motifs affected by exon skipping in Tnpo3-deficient mouse CD4+ thymocytes.

Ensembl Gene ID	Gene
**Possible TNPO3 cargo**	
ENSMUSG00000018379	*Srsf1*
ENSMUSG00000071172	*Srsf3*
ENSMUSG00000028911	*Srsf4*
ENSMUSG00000016921	*Srsf6*
ENSMUSG00000055436	*Srsf11*
ENSMUSG00000022983	*Scaf4(Srsf15)*
ENSMUSG00000022119	*Rbm26*
ENSMUSG00000024491	*Rbm27*
ENSMUSG00000006498	*Ptbp1*
ENSMUSG00000046201	*Scaf8*
**Other splice regulators**	
ENSMUSG00000028382	*Ptbp3*
ENSMUSG00000078765	*U2af1l4*
ENSMUSG00000028820	*Sfpq*
ENSMUSG00000020358	*Hnrnpab*
ENSMUSG00000029328	*Hnrnpdl*
ENSMUSG00000045427	*Hnrnph2*
ENSMUSG00000059208	*Hnrnpm*
ENSMUSG00000004980	*Hnrnpa2b1*
ENSMUSG00000015165	*Hnrnpl*
ENSMUSG00000024095	*Hnrnpll*
ENSMUSG00000007850	*Hnrnph1*
ENSMUSG00000032580	*Rbm5*
ENSMUSG00000041815	*Poldip3*
ENSMUSG00000030846	*Tial1*
ENSMUSG00000005506	*Celf1*
**Other RNA-binding proteins**	
ENSMUSG00000009079	*Ewsr1*
ENSMUSG00000042396	*Rbm7*
ENSMUSG00000048271	*Rbm33*
ENSMUSG00000033931	*Rbm34*
ENSMUSG00000021938	*Pspc1*
ENSMUSG00000034681	*Rnps1*
ENSMUSG00000025571	*Tnrc6c*
ENSMUSG00000030016	*Zfml*
ENSMUSG00000037236	*Matr3*
ENSMUSG00000032212	*Sltm*
ENSMUSG00000071337	*Tia1*
ENSMUSG00000028898	*Trnau1ap*
ENSMUSG00000069769	*Msi2*
ENSMUSG00000069565	*Dazap1*

Supplementary Table 2 Primers used in this study.

	Forward primer		Reverse primer		Primer position	Splicing status of amplicon
qPCR						
Trav11 gene						
	Hprt-L	TCCTCCTCAGACCGCTTTT	Hprt-R	CCTGGTTCATCATCGCTAATC	Hprt exon1 - exon2	
	musTrav11-L21	GAGGCAGGCTTCTCCAGAAC	musTrav11-R31	ACTGATAATGCAGCCACAAGACC	endogenous Trav11 5'UTR (exon1) - Trav11 (exon1)	spliced + unspliced Trav11
	musTrav11-L21	GAGGCAGGCTTCTCCAGAAC	musTrav11-R32	GTCTTGCCAGCCACCCACTG	endogenous Trav11 5'UTR (exon1) - Trav11 (exon1+2)	spliced Trav11
	musTrav11-L21	GAGGCAGGCTTCTCCAGAAC	musTrav11int-R1	CCCGGCTAATATCTCCACTC	endogenous Trav11 5'UTR (exon1) - Trav11 intron	unspliced Trav11
	musTrav11TG-L1	GCTTGAATCCCACGATTCGG	musTrav11-R31	ACTGATAATGCAGCCACAAGACC	pLck promoter - Trav11 (exon1)	spliced + unspliced Trav11
	musTrav11TG-L1	GCTTGAATCCCACGATTCGG	musTrav11-R32	GTCTTGCCAGCCACCCACTG	pLck promoter - Trav11 (exon1+2)	spliced Trav11
	musTrav11TG-L1	GCTTGAATCCCACGATTCGG	musTrav11int-R1	CCCGGCTAATATCTCCACTC	pLck promoter - Trav11 intron	unspliced Trav11
	Trav11-10	gatgagaactccaagtactagtgc	Traj18_2	cctggagtagaaagaaacctac	cctggagtagaaagaaacctac	unspliced Trav11-Traj18-Trac
Other genes						
	Hnrnpa1_f	ctctgtcgaagcaagagatg	Hnrnpa1_r	aaccgcctccacgaccaccac		
	Zbtb16_f	gcgactgagaatgcatttactggc	Zbtb16_r	gggtctgtctgtgtgtctccag		
	Myb_f	ctccatctcagctctc	Myb_r	ggtcttcgtcgttatagtg		
RT-PCR						
	Mus-GAPDH-L	CCGGTGCTGAGTATGTCGTG	Mus-GAPDH-R	CAGTCTTCTGGGTGGCAGTG	Gapdh exon1 - exon3	
	musTrav11-L21	GAGGCAGGCTTCTCCAGAAC	musTRAC-R4	CAGGTTCTGGGTTCTGGATG	endogenous Trav11 5'UTR (exon1) - Trac	
