# Supplementary material for: Tnpo3 controls splicing of the pre-mRNA encoding the canonical TCR α chain of iNKT cells
Source: Nat Commun. 2023 Jun 20;14:3645. doi: 10.1038/s41467-023-39422-4 (PMC10282035; doi:10.1038/s41467-023-39422-4)
Supplement: Supplementary file 2 — Description of Additional Supplementary Files [file 41467_2023_39422_MOESM2_ESM.pdf]

Description of Additional Supplementary Files

Supplementary Data 1. Alternative splicing events (exon skipping) associated with *Tnpo3*-deficiency at a False Discovery Rate (FDR) threshold of 1% (Sheet 1, $CD4^+$ single-positive T cells; sheet 2, $CD4^+CD8^+$ double-positive T cells). Chromosome, strand, and skipped exon start and end denote the coordinates of the spliced-out exon. FDR denotes the false discovery rate of the differential alternative splicing event. Inclusion level denotes the mean exon inclusion level for either wild-type or mutant. Δ Inclusion level denotes the difference in inclusion level between wild-type and mutant.
