# Supplementary material for: Tnpo3 controls splicing of the pre-mRNA encoding the canonical TCR α chain of iNKT cells
Source: Nat Commun. 2023 Jun 20;14:3645. doi: 10.1038/s41467-023-39422-4 (PMC10282035; doi:10.1038/s41467-023-39422-4)
Supplement: Supplementary file 4 — Reporting Summary [file 41467_2023_39422_MOESM4_ESM.pdf]

nature portfolio

Corresponding author(s): Thomas Boehm

Last updated by author(s): Jun 5, 2023

Reporting Summary

Nature Portfolio wishes to improve the reproducibility of the work that we publish. This form provides structure for consistency and transparency in reporting. For further information on Nature Portfolio policies, see our Editorial Policies and the Editorial Policy Checklist.

Statistics

For all statistical analyses, confirm that the following items are present in the figure legend, table legend, main text, or Methods section.

n/a	Confirmed	
☐	☒	The exact sample size (*n*) for each experimental group/condition, given as a discrete number and unit of measurement
☐	☒	A statement on whether measurements were taken from distinct samples or whether the same sample was measured repeatedly
☐	☒	The statistical test(s) used AND whether they are one- or two-sided<br>*Only common tests should be described solely by name; describe more complex techniques in the Methods section.*
☒	☐	A description of all covariates tested
☐	☒	A description of any assumptions or corrections, such as tests of normality and adjustment for multiple comparisons
☐	☒	A full description of the statistical parameters including central tendency (e.g. means) or other basic estimates (e.g. regression coefficient) AND variation (e.g. standard deviation) or associated estimates of uncertainty (e.g. confidence intervals)
☐	☒	For null hypothesis testing, the test statistic (e.g. *F*, *t*, *r*) with confidence intervals, effect sizes, degrees of freedom and *P* value noted<br>*Give P values as exact values whenever suitable.*
☒	☐	For Bayesian analysis, information on the choice of priors and Markov chain Monte Carlo settings
☒	☐	For hierarchical and complex designs, identification of the appropriate level for tests and full reporting of outcomes
☒	☐	Estimates of effect sizes (e.g. Cohen's *d*, Pearson's *r*), indicating how they were calculated

Our web collection on statistics for biologists contains articles on many of the points above.

Software and code

Policy information about availability of computer code

Data collection	BD FACSDiva software (up to version 6.1.3 was used for data collection;
Data analysis	RNA-seq data. (a) Alignment of RNA-seq reads. The raw RNA-seq reads were mapped against the mouse genome assembly GRCm38 with the spliced read aligner TopHat2 version 2.0.13 (ref. 68). Reads were first mapped to the transcriptome provided by the mouse gene annotation of GENCODE release M4 (ref. 69). Unmapped reads were subsequently aligned against the mouse genome. TopHat2 was run with the library type parameter "fr-secondstrand" as the RNA-seq library was prepared with a strand-specific protocol. Strand-specific library type, fragment length mean and standard deviation were determined on a subsample of 1 million reads with RSeQC version 2.4 (ref. 70), and provided to TopHat2. All other TopHat2 parameters were set to default. (b) Analysis of differential gene expression and exon usage. The numbers of sequenced fragments per annotated gene (based on GENCODE M4) were quantified with featureCounts version 1.4.5-p1 (ref. 71). Fragments were only counted if both ends aligned on the same chromosome with a minimum mapping quality of 10 and in agreement with the strand orientation of the annotated gene. Reads per exons were counted with HTSeq version 0.6.1 (ref. 72) using analogous parameters, but including only transcripts with GENCODE tag "basic". Differential gene expression analysis was performed with the Bioconductor package edgeR version 3.12.0 (ref. 73). Weakly expressed genes with less than 0.2 counts-per-million (CPM) in at least 3 samples were filtered out. Dispersion estimation and testing for differential expression between heterozygous control (Tnpo3+/fl; pLck:Cre) and homozygous mutant (Tnpo3fl/fl; pLck:Cre) samples were performed using the quasi-likelihood (QL) method74, robustified against potential outlier genes. Exon read counts were normalized with the Bioconductor package DEXSeq version 1.16.0 (ref. 75). The exon expression scatter plot shows the mean read counts, normalized by sample-specific size factors, per exon for genotypes Tnpo3 heterozygous (Tnpo3+/fl; pLck:Cre) vs. homozygous (Tnpo3fl/fl; pLck:Cre) mutant mice. The exon usage of alternative transcript isoforms is visualized by Sashimi plots76. (c) Analysis of differential alternative splicing events. The BAM files with TopHat2 aligned reads were filtered to include only reads that overlap with annotated transcripts in the correct strand orientation. The filtered BAM files for both genotypes were then used as input for the differential alternative splicing (AS) analysis with rMATS version 3.0.9 (ref. 77). Of all reads, rMATS only considers unique, properly paired reads that are

mapped without insertions and deletions. The mean fragment size of the paired-end samples and its standard deviation were determined as described above and provided to rMATS. All other parameters were set to default. Based on the provided GENCODE M4 annotation, rMATS builds a database of possible AS events that are either (i) already annotated by alternative gene isoforms or (ii) novel and supported by reads covering un-annotated exon junctions between annotated exons within a gene. The reported AS events are of the following types: exon skipping, retained intron, mutually exclusive exons, alternative 5' donor or 3' acceptor splice sites. We selected for each type all AS events with at least 5% difference in the mean exon inclusion levels that were identified by reads spanning splice junctions at 1% FDR. Functional enrichment analysis of genes with significant AS events was performed with the DAVID Functional Annotation Clustering tool78,79. For each set of genes with significant AS events of a specific type (e.g. exon skipping), the genes were tested for functional enrichment by comparing them to the background of all genes that were tested for the respective AS event type. (d) Quantification of Tcra and Tcrb transcript levels. The abundance of spliced and unspliced transcripts was quantified with Salmon version 0.4.2 (ref. 80). The index included all spliced and unspliced Tcra and Tcrb transcripts based on GENCODE M4 annotation, excluding pseudogenes and Trcra gene duplicates (identified by trailing “d” or “n” in gene name). Salmon was called in extra-sensitive search mode and with 98% required read coverage. The beta-binomial test81 was used to test for significant differences in the splicing rate and the relative abundance of transcripts between genotypes; P values were adjusted for multiple testing by Bonferroni correction.

FloJo software (up to version 10.9.0) was used for data analysis; statistical analysis was performed with GraphPad Prism (up to version 9.5.1) software.

For manuscripts utilizing custom algorithms or software that are central to the research but not yet described in published literature, software must be made available to editors and reviewers. We strongly encourage code deposition in a community repository (e.g. GitHub). See the Nature Portfolio guidelines for submitting code & software for further information.

Data

Policy information about availability of data

All manuscripts must include a data availability statement. This statement should provide the following information, where applicable:

- Accession codes, unique identifiers, or web links for publicly available datasets
- A description of any restrictions on data availability
- For clinical datasets or third party data, please ensure that the statement adheres to our policy

All data needed to evaluate the conclusions in the paper are present in the paper or the Supplementary Materials. RNA-Seq data are deposited in NCBI´s Gene Expression Omnibus under GEO accession number GSE77137 (https://www.ncbi.nlm.nih.gov/geo/query/acc.cgi?acc=GSE77137). The data in Fig. 5d were taken from publicly available sources (GSE37448; https://www.ncbi.nlm.nih.gov/geo/query/acc.cgi?acc=GSE37448). Source data are provided with this paper.

Human research participants

Policy information about studies involving human research participants and Sex and Gender in Research.

Reporting on sex and gender	not applicable
Population characteristics	not applicable
Recruitment	not applicable
Ethics oversight	not applicable

Note that full information on the approval of the study protocol must also be provided in the manuscript.

Field-specific reporting

Please select the one below that is the best fit for your research. If you are not sure, read the appropriate sections before making your selection.

- [x] Life sciences
- [ ] Behavioural & social sciences
- [ ] Ecological, evolutionary & environmental sciences

For a reference copy of the document with all sections, see nature.com/documents/nr-reporting-summary-flat.pdf

Life sciences study design

All studies must disclose on these points even when the disclosure is negative.

Sample size	The sample size for each experiment is indicated in the figure legends. Sample sizes were based on our experience and common practice in the related fields, balancing statistic robustness, resource availability and animal welfare. No Statistical methods were used to predetermine sample size.
Data exclusions	No data were excluded.
Replication	Several biological replicates were included in the study design;the results of replicate experiments were in agreement. The number of biological replicates ranged from 2-7.

Randomization	Randomization was not performed, as the animals had to be selected for analysis based on the appropriate genotypes.
Blinding	The experimenters were not blinded as to the identity of the biological samples; as the phenotype of mutant and transgenic mice is obvious during the analysis of individual mice, blinding is not feasible.

Reporting for specific materials, systems and methods

We require information from authors about some types of materials, experimental systems and methods used in many studies. Here, indicate whether each material, system or method listed is relevant to your study. If you are not sure if a list item applies to your research, read the appropriate section before selecting a response.

Materials & experimental systems

n/a	Involved in the study	
☐	☒	Antibodies
☒	☐	Eukaryotic cell lines
☒	☐	Palaeontology and archaeology
☐	☒	Animals and other organisms
☒	☐	Clinical data
☒	☐	Dual use research of concern

Methods

n/a	Involved in the study	
☒	☐	ChIP-seq
☐	☒	Flow cytometry
☒	☐	MRI-based neuroimaging

Antibodies

Antibodies used

FITC-conjugated anti-CD4 (clone GK1.5; BioLegend Cat# 100405; 1:1000 dilution); PE-conjugated anti-CD8 (clone 53-6.7; eBioscience Cat #12-0081-82; 1:800 dilution); PerCP-Cy5.5-conjugated anti-CD3 (clone 145-2C11; Biolegend Cat# 100327; 1:100 dilution); APC-conjugated anti-CD19 (clone MB19-1; eBioscience Cat#17-0191-82; 1:100 dilution); PE-conjugated CD1d Tetramer (Proimmune Cat# D001-2X; 1:200 dilution); FITC-conjugated anti-TCR (clone eBioGL3; eBioscience Cat#11-5711-82; 1:100 dilution); PE-conjugated anti-TCR (clone H57-597; eBioscience Cat#12-5961-82; 1:300 dilution). For preparative flow cytometry, the following antibodies were used: APC-Cy7-conjugated anti-CD4 (clone GK1.5; Biolegend Cat#100413; 1:400 dilution); FITC-conjugated anti-CD8a (clone 53-6.7; eBioscience Cat#11-0081-82; 1:800 dilution); PerCP-Cy5.5-conjugated anti-TCR (clone H57-597; eBioscience Cat#45-5961-82; 1:100 dilution); PE-Cy7-conjugated anti-CD24 (clone M1/69; eBioscience Cat#15-0242-82; 1:1000); PE-conjugated CD1d Tetramer (Proimmune Cat# D001-2X; 1:400 dilution).

Validation

PE-conjugated CD1d Tetramer (https://www.proimmune.com/wp-content/uploads/2021/02/PS_DE001-RPE_V1.3-CD1d-Tetramer-%CE%B1-GalCer-Loaded-R-PE-Labeled.pdf)

FITC-conjugated anti-CD4 (https://www.biolegend.com/en-us/products/fitc-anti-mouse-cd4-antibody-248?pdf=true&displayInline=true&leftRightMargin=15&topBottomMargin=15&filename=FITC%20anti-mouse%20CD4%20Antibody.pdf&v=20220421053143)

PerCP-Cy5.5-conjugated anti-CD3 (https://www.biolegend.com/en-us/products/percp-cyanine5-5-anti-mouse-cd3epsilon-antibody-4191?pdf=true&displayInline=true&leftRightMargin=15&topBottomMargin=15&filename=PerCP/Cyanine5.5%20anti-mouse%20CD3%CE%B5%20Antibody.pdf&v=20230114013553)

PE-conjugated anti CD8a (https://www.thermofisher.com/antibody/product/CD8a-Antibody-clone-53-6-7-Monoclonal/12-0081-82)

APC-conjugated anti-CD19 (https://www.thermofisher.com/antibody/product/CD19-Antibody-clone-MB19-1-Monoclonal/17-0191-82)

FITC-conjugated anti-TCRgammadelta (https://www.thermofisher.com/antibody/product/TCR-gamma-delta-Antibody-clone-eBioGL3-GL-3-GL3-Monoclonal/11-5711-82)

PE-conjugated anti-TCR (https://www.thermofisher.com/antibody/product/TCR-beta-Antibody-clone-H57-597-Monoclonal/12-5961-82)

PerCP-Cy5.5-conjugated anti- TCR (https://www.thermofisher.com/antibody/product/TCR-beta-Antibody-clone-H57-597-Monoclonal/45-5961-82)

FITC-conjugated anti-CD8a(https://www.thermofisher.com/antibody/product/CD8a-Antibody-clone-53-6-7-Monoclonal/11-0081-82)

PE-Cy7-conjugated anti-CD24 (https://www.thermofisher.com/antibody/product/CD24-Antibody-clone-M1-69-Monoclonal/15-0242-82)

APC-Cy7-conjugated anti-CD4 (https://www.biolegend.com/en-us/products/apc-cyanine7-anti-mouse-cd4-antibody-1964)

Animals and other research organisms

Policy information about studies involving animals; ARRIVE guidelines recommended for reporting animal research, and Sex and Gender in Research

Laboratory animals	The generation of Tnpo3 conditional knock-out mice was achieved as follows. The ES cell line EPD0318_3_G02 (genetic background: C57BL/6N Agouti(A/a); allele name: Tnpo3tm1a(KOMP)Wtsi) was obtained from the KOMP Repository and used to derive chimaeric mice using standard procedures. Chimaeras were crossed with mice constitutively expressing FLP recombinase (B6:SJL-Tg(ACTFLPe)9205Dym/J [Jackson Laboratory stock number 003800])63 to remove the lacZ/neo cassette which is flanked by FRT sites. As a result, the ES-derived allele of Tnpo3 possesses an exon 7 flanked by loxP sites. Constitutive deletion of exon 7 was achieved by crossing heterozygous mice with mice with a universal Cre-deleter strain (B6.C-Tg(CMV-cre)1Cgn/J [Jackson Laboratory stock number 006054])64. Tnpo3 deficiency is embryonic lethal. T cell-specific deletion was achieved by crosses with mice transgenic for an pLck:Cre construct52. Wild-type and floxed alleles of Tnpo3 were amplified from genomic DNA using primers 5´-GGAATTCAGTGCTCTGTACC and 5´-TCCAGCTCGGGATCCAATGC (amplicon sizes: wild-type allele, 227 bp; floxed allele, 342 bp); the deleted allele was amplified using primers 5´-GGAATTCAGTGCTCTGTACC and 5´-CAATTCCTGAAGCCACCCTG (amplicon sizes: deleted allele, 266 bp; wild-type allele, 947 bp; floxed allele, 1,072 bp). Partial Tnpo3 cDNAs were amplified using primers 5´-CGAAGCTGCTTCAGACTGTG (located in exon 6) and 5´-ATGTTCTCCTAGTCGGTACC (located in exon 8); amplicon size of the wild-type form, 331 bp; of the mutant form, 192 bp. The mouse floxed Tnpo3 allele (44) was maintained on the C57BL/6J background.
Wild animals	No wild animals were used
Reporting on sex	Mice of both sexes were used; no differences in phenotypes were observed
Field-collected samples	No field-collected samples were used
Ethics oversight	All animal experiments were approved by the institute's review committee and conducted under license from and approved by the local government. Ethics committee: Regierungspräsidium Freiburg (license 35-9185.81/G-15/35).

Note that full information on the approval of the study protocol must also be provided in the manuscript.

Flow Cytometry

Plots

Confirm that:

- [x] The axis labels state the marker and fluorochrome used (e.g. CD4-FITC).
- [x] The axis scales are clearly visible. Include numbers along axes only for bottom left plot of group (a 'group' is an analysis of identical markers).
- [x] All plots are contour plots with outliers or pseudocolor plots.
- [x] A numerical value for number of cells or percentage (with statistics) is provided.

Methodology

Sample preparation	Thymocyte and splenocytesuspensions were prepared in parallel by mechanical liberation, achieved by gently pressing thymic lobes through 40 µm sieves.
Instrument	BD Fortessa II; MoFlow; both from Dako Cytomation-Beckman Coulter
Software	D FACSDiva software (up to version 6.1.3 was used for data collection; FloJo software (up to version 10.9.0) was used for data analysis; statistical analysis was performed with GraphPad Prism (up to version 9.5.1) software.
Cell population abundance	Purity was determined by running a purity check of the sorted populations after the sort was completed.
Gating strategy	All samples were initially gated using forward and side scatter to identify events corresponding to cells, doublets are excluded by gating on single cells using forward scatter height vs. area, alive cells were selected by negativity for the viability dye Fluoro Gold, the follow gaiting steps are according to the marker genes described in the manuscript.

- [x] Tick this box to confirm that a figure exemplifying the gating strategy is provided in the Supplementary Information.
